# Supplementary material for: Proteasomal degradation induced by DPP9‐mediated processing competes with mitochondrial protein import
Source: EMBO J. 2020 Aug 20;39(19):e103889. doi: 10.15252/embj.2019103889 (PMC7527813; doi:10.15252/embj.2019103889)
Supplement: Supplementary file 1 — Appendix [file EMBJ-39-e103889-s001.pdf]

Appendix

DPP9-processing-induced proteasomal degradation competes with mitochondrial protein import

Yannik Finger[1,*], Markus Habich[1,*], Sarah Gerlich[1], Sophia Urbanczyk[2], Erik van de Logt[1], Julian Koch[1], Laura Schu[1], Kim Jasmin Lapacz[1], Muna Ali[1], Carmelina Petrungaro[1], Silja Lucia Salscheider[1], Christian Pichlo[3], Ulrich Baumann[3], Dirk Mielenz[2] Joern Dengjel[4], Bent Brachvogel[5,6], Kay Hofmann[7], Jan Riemer[1,8]

1, Institute of Biochemistry, Redox Biochemistry, University of Cologne, Zuelpicher Str. 47a/R. 3.49, 50674 Cologne, Germany
2, Division of Molecular Immunology, Department of Internal Medicine III, Nikolaus-Fiebiger-Center, University of Erlangen-Nürnberg, Erlangen, Germany
3, Institute of Biochemistry, University of Cologne, Zuelpicher Str. 47, 50674 Cologne, Germany
4, Department of Biology, University of Fribourg, Chemin du Musée 10, 1700 Fribourg, Switzerland
5, Department of Pediatrics and Adolescent Medicine, Experimental Neonatology, Faculty of Medicine, University of Cologne, Cologne, Germany
6, Center for Biochemistry, Faculty of Medicine, University of Cologne, Cologne, Germany.
7, Institute of Genetics, University of Cologne, Zuelpicher Str. 47a, 50674 Cologne, Germany
8, Cologne Excellence Cluster on Cellular Stress Responses in Aging-Associated Diseases (CECAD), University of Cologne, 50931Cologne, Germany.

*, both authors contributed equally
Address correspondence to: JR, jan.riemer@uni-koeln.de, phone: +49-221-470-7306

This Appendix includes:

- Table S1. Plasmids, cell lines, siRNAs, and primers used in this study.
- Figures S1-S7

Table S1. Plasmids, cell lines, siRNAs, and primers used in this study.

Cell line	Plasmid	Gene	Tag (N-terminal)	Tag (C-terminal)	Reference
HeLa	-	-		-	
Flp-In T-REx-293-Mock	pcDNA5/FRT/TO	--	--	--	Fischer et al., 2013
Flp-In T-REx-293-MIA40	pcDNA5/FRT/TO	ORF *MIA40*	--	Strep	Fischer et al., 2013
Flp-In T-REx-293-MIA40 C53S	pcDNA5/FRT/TO	ORF *MIA40 (T157►G)*	--	Strep	Habich et al., 2019
Flp-In T-REx-293-MIA40 C55S	pcDNA5/FRT/TO	ORF *MIA40 (T163►G)*	--	Strep	Habich et al., 2019
Flp-In T-REx-293-MIA40 C53S,C55S	pcDNA5/FRT/TO	ORF *MIA40 (T157; T163►G)*	--	Strep	Fischer et al., 2013
Flp-In T-REx293-NDUFB10	pcDNA5/FRT/TO	ORF *NDUFB10*	--	HA	Habich et al., 2019
Flp-In T-REx293-NDUFB10 C107S	pcDNA5/FRT/TO	ORF *NDUFB10* T_{319}►*A*	--	HA	Habich et al., 2019
Flp-In T-REx293-AK2	pcDNA5/FRT/TO	ORF *AK2*	--	HA	this study
Flp-In T-REx293-AK2 A2D	pcDNA5/FRT/TO	ORF *AK2* C5►A	--	HA	this study
Flp-In T-REx293-AK2 S4P	pcDNA5/FRT/TO	ORF *AK2* A10►C; G11►C	--	HA	this study
Flp-In T-REx293-AK2 K28Q	pcDNA5/FRT/TO	ORF *AK2* A82►C	--	HA	this study
Flp-In T-REx293-AK2 C40S,C42S,C92S	pcDNA5/FRT/TO	ORF *AK2* G119►C; G125►C; G275►C	--	HA	this study
Flp-In T-REx293-AK2 A2D,C40S,C42S,C92S	pcDNA5/FRT/TO	ORF *AK2* C5►A; G119►C; G125►C; G275►C	--	HA	this study
Flp-In T-REx293-AK2 S4P,C40S,C42S,C92S	pcDNA5/FRT/TO	ORF *AK2* A10►C; G11►C; G119►C; G125►C; G275►C	--	HA	this study
Flp-In T-REx293-AK2 S4G,C40S,C42S,C92S	pcDNA5/FRT/TO	ORF *AK2* A10►G; G119►C; G125►C; G275►C	--	HA	this study
Flp-In T-REx293-AK2 S4V,C40S,C42S,C92S	pcDNA5/FRT/TO	ORF *AK2* A10►G; G11►T; G119►C; G125►C; G275►C	--	HA	this study
Flp-In T-REx293-AK2 K28Q,C40S,C42S,C92S	pcDNA5/FRT/TO	ORF *AK2* A82►C; G119►C; G125►C; G275►C	--	HA	this study
Flp-In T-REx293-AK2 C40S	pcDNA5/FRT/TO	ORF *AK2* G119►C	--	HA	this study
Flp-In T-REx293-AK2 C42S	pcDNA5/FRT/TO	ORF *AK2* G125►C	--	HA	this study
Flp-In T-REx293-AK2 C92S	pcDNA5/FRT/TO	ORF *AK2* G275►C	--	HA	this study
Flp-In T-REx293-AK2 C232S	pcDNA5/FRT/TO	ORF *AK2* G695►C	--	HA	this study
Flp-In T-REx293-CHCHD2	pcDNA5/FRT/TO	ORF *CHCHD2*	--	HA	this study
Flp-In T-REx293-CHCHD2 P2L	pcDNA5/FRT/TO	ORF *CHCHD2* C5►T	--	HA	this study
Rosetta2 (DE3)-AK2 C40,232S	pRSET-A	ORF *AK2* G119►C; G695►C	6xHis	--	this study
Rosetta2 (DE3)-AK2 C40, 42, 92, 232S	pRSET-A	G119►C; G125►C; G275►C; G695►C	6xHis	--	this study
Rosetta2 (DE3)-AK1	pQTEV	ORF *AK1*	7xHis	--	Bussow et al., 2005

Primer	Sequence (5’-3’)	Restriction site
MIA40 fwd. for pcDNA5/FRT/TO	CCAAGCTTGCCATGTCCTATTGCCGG	*HindIII*
MIA40 rev. for pcDNA5/FRT/TO	CCCTCGAGTTATTTCTCAAATTGTGGATGACTCCATCC TCCAGCACTTGATCCCTCCTCTTCTTTG	*XhoI*
MIA40 fwd. (T157►A157) for pcDNA5/FRT/TO	GGAAACATTAACTGGAACAGCCCATGCCTTGGGGG	
MIA40 rev. (T157►A157) for pcDNA5/FRT/TO	CCCCCAAGGCATGGGCTGTTCCAGTTAATGTTTCC	
MIA40 fwd. (T163►A163) for pcDNA5/FRT/TO	AACTGGAACTGCCCAAGCCTTGGGGGAATGGCC	
MIA40 rev. (T163►A163) for pcDNA5/FRT/TO	GGCCATTCCCCCAAGGCTTGGGCAGTTCCAGTT	
MIA40 fwd. (T202;T204►G; T203►A) for pcDNA5/FRT/TO	CCTGTGGAGAACAGGAGAAGTCAGCCTTTTCC	
MIA40 rev. (T202;T204►G; T203►A) for pcDNA5/FRT/TO	GGAAAAGGCTGACTTCTCCTGTTCTCCACAGG	
NDUFB10 fwd. for pcDNA5/FRT/TO	GGGGTACCGCCATGCCGGACAGCTGGGAC	*KpnI*
NDUFB10 rev. for pcDNA5/FRT/TO	CCGGATCCCTAAGCGTAATCTGGAACATCGTATGGGT ATCCACTTCCAGTGAGCACAAGGGAGGGG	*BamHI*
NDUFB10 fwd. (T319►A) for pcDNA5/FRT/TO	GGATCGGCTCAAAGCCAGTCAGCAGAGGGAAGG	
NDUFB10 rev. (T319►A) for pcDNA5/FRT/TO	CCTTCCCTCTGCTGACTGGCTTTGAGCCGATCC	
AK2 fwd. for pcDNA5/FRT/TO	CCGGTACCTCCATGGCTCCCAGCGTGC	*KpnI*
AK2 rev. for pcDNA5/FRT/TO	CGGCGGCCGCTTATGCATAATCAGGTACATCATAAGG ATAGATAAACATAACCAAGTCTTTACATGTG	*NotI*
AK2 fwd. (G119►C; G125►C) for pcDNA5/FRT/TO	GGCTGAAAACTTCTCTGTCTCCCATTTAGCTACTGGG	
AK2 rev. (G119►C; G125►C) for pcDNA5/FRT/TO	CCCAGTAGCTAAATGGGAGACAGAGAAGTTTTCAGCC	
AK2 fwd. (G275►C) for pcDNA5/FRT/TO	GGAGACCCCCTTGTCCAAAAATGGTTTTCTTCTGG	
AK2 rev. (G275►C) for pcDNA5/FRT/TO	CCAGAAGAAAACCATTTTTGGACAAGGGGGTCTCC	
AK2 fwd. (A82►C) for pcDNA5/FRT/TO	GGGGCCGGTCAAGGGACCCAGG	
AK2 rev. (A82►C) for pcDNA5/FRT/TO	CCTGGGTCCCTTGACCGGCCCC	
AK2 fwd. (C5►A) for pcDNA5/FRT/TO	CCGGTACCGACATGGATCCCAGCGTGCCAGC	*KpnI*
AK2 fwd. (A10►C; G11►C) for pcDNA5/FRT/TO	CCGGTACCGACATGGCTCCCCCCGTGCCAGC	*KpnI*
AK2 fwd. (A10►G; G11►T) for pcDNA5/FRT/TO	CCGGTACCGACATGGCTCCCGTCGTGCCAGC	*KpnI*
AK2 fwd. (A10►G) for pcDNA5/FRT/TO	CCGGTACCGACATGGCTCCCGGCGTGCCAGC	*KpnI*

siRNA	Source	Identifier
Ctrl	*Qiagen*	SI03650318
PSMB3	*Qiagen*	SI02654239
PSMC5	*Qiagen*	SI00301476
DPP8	*Qiagen*	SI02642864<br>SI00149611
DPP9	*Qiagen*	SI02757853<br>SI02757860
MIA40	*Qiagen*	SI04292176

Antibodies, reagents, resources	Source	Identifier
**Antibodies**		
Mouse monoclonal anti-Strep	Abcam	Cat# ab184224
Rabbit polyclonal anti-HA	Sigma-Aldrich	Cat# SAB4300603; RRID:AB_10620829
Mouse monoclonal anti-HA, agarose conjugate	Sigma-Aldrich	Cat#A2095 RRID: AB_257974
Rat monoclonal anti-HA (used for immunofluorescence)	Roche	Cat#11867423001
Mouse monoclonal anti-cytochrome c	BD-Biosciences	Cat# 556432; RRID:AB_396416
Mouse monoclonal anti-LDH (H-10)	Santa-Cruz	Cat# sc-133123; RRID:AB_2134964
Rabbit polyclonal anti-COX17	Biorbyt	Cat# orb160552
Rabbit polyclonal anti-COX6B1	Sigma-Aldrich	Cat# HPA004192, RRID:AB_1847180
Rabbit polyclonal anti-DPP8	Sigma	Cat# HPA008706
Rabbit polyclonal anti-DPP9	Abcam	Cat# ab42080
Rabbit polyclonal anti-NDUFB7	Abcam	Cat# ab188575
Rabbit polyclonal anti-NDUFS5	Abcam	Cat# ab179806
Rabbit polyclonal anti-NDUFA8	Abcam	Cat# ab184952
Rabbit polyclonal anti-CHCHD6	Sigma-Aldrich	Cat# HPA047673; RRID:AB_10961761
Rabbit polyclonal anti-CMC1	Sigma-Aldrich	Cat# HPA043333; RRID:AB_10797139
Rabbit polyclonal anti-NDUFB10	Abcam	Cat# ab196019
Rabbit polyclonal anti-CHCHD3	Abcam	Cat# ab154500
Mouse monoclonal anti-HSP70	Santa-Cruz	Cat# sc-66048; RRID:AB_832518
Mouse monoclonal anti-TIMM8A	Santa-Cruz	Cat# sc-101282; RRID:AB_2204692
Mouse monoclonal anti-TIMM9	Santa-Cruz	Cat# sc-101285; RRID:AB_2287477
Mouse monoclonal anti-AK1	Santa-Cruz	Cat# sc-365048 RRID: AB_10842152
Rabbit polyclonal anti-AK2	Self-made	this study
Rabbit polyclonal anti-SMAC	Sigma-Aldrich	Cat# SAB3500327; RRID:AB_10638009
Mouse monoclonal anti-PDH	Santa-Cruz	Cat# sc-377092; RRID:AB_2716767
Mouse monoclonal anti-GAPDH	Santa-Cruz	Cat# sc-32233; RRID:AB_627679
Rabbit polyclonal anti-COX19	Fischer et al., 2013	N/A
Rabbit polyclonal anti-ALR	Fischer et al., 2013	N/A
Rabbit polyclonal anti-MIA40	Fischer et al., 2013	N/A
Rabbit polyclonal anti-CCS1	Suzuki et al, 2013	N/A
Mouse monoclonal anti-Histone H3	Sigma-Aldrich	Cat# SAB4200651
Rabbit polyclonal anti-CPOX	St. John's Laboratory	Cat# STJ23214
Rabbit polyclonal anti-CHCHD2	Proteintech	Cat# 19424-1-AP RRID:AB_10638907
Goat anti-Mouse - Affinity Pure, HRP Conjugate	ImmunoReagents	Cat# GtxMu-003-DHRPX
Goat anti-Rabbit - Affinity Pure, HRP Conjugate	ImmunoReagents	Cat# GtxRb-003-DHRPX
Goat anti-Rat, Alexa Fluor 488	Invitrogen	Cat# A-11006; RRID:AB_2534074
Goat anti-Mouse, Alexa Fluor 594	Invitrogen	Cat# A-31623
Bacterial and Virus Strains		
One Shot™ TOP10 Chemically Competent E. coli	Thermo Fisher	Cat# C404010
Rosetta™ 2 (DE3) Singles™ Competent Cells	Novagen	Cat#71400-3

**Chemicals, Peptides, and Recombinant Proteins**		
MG132 (Z-Leu-Leu-Leu-al)	Sigma-Aldrich	Cat #C2211; CAS: 133407-82-6
1G244 (2-Amino-4-{4-[bis(4-fluorophenyl)methyl]piperazin-1-yl}-1-(2,3-dihydro-1H-isoindol-2-yl)butane-1,4-dione)	AK Scientific	Cat#Y0432
Methyl-PEG-Maleimide, mmPEG12	ThermoFisher	Cat#22711
Tris(2-carboxyethyl)phosphine hydrochloride	Sigma-Aldrich	Cat#C4706
β-Nicotinamide adenine dinucleotide, reduced	Sigma-Aldrich	Cat#N8129
Phospho(enol)pyruvic acid	Sigma-Aldrich	Cat#P0564
Adenosine 5'-triphosphate	Sigma-Aldrich	Cat#10127523001
Adenosine 5'-diphosphate	Sigma-Aldrich	Cat#A2754
Adenosine 5'-monophosphate	Sigma-Aldrich	Cat#A2252
β-Nicotinamide adenine dinucleotide phosphate	Sigma-Aldrich	Cat#N0505
SigmaFast protease inhibitor tablets	Sigma-Aldrich	Cat#S8820
Critical Commercial Assays		
EasyTag EXPRESS 35S Protein Labeling Mix	Perkin-Elmer	Cat# NEG772
PrestoBlue Cell Viability Reagent	Thermo Fisher	Cat# A13261
TnT Quick Coupled Transcription/Translation System	Promega	Cat# L1170
Pierce 660nm Protein Assay Reagent	Thermo Scientific	Cat# 22660
Hexokinase/Glucose-6-Phosphate Dehydrogenase	Sigma-Aldrich	Cat#10737275001
Pyruvate Kinase/Lactic Dehydrogenase	Sigma-Aldrich	Cat#P0294
**Software and Algorithms**		
SWISS-MODEL	Waterhouse et al., 2018	https://swissmodel.expasy.org/
CellProfiler 3.1.5	Carpenter et al., 2006	https://cellprofiler.org/
Jalview 2.8.2	Waterhouse et al., 2009	http://www.jalview.org/
PyMOL 1.8.7.0	Schrodinger, LLC	
Image Lab 5.2	Biorad Laboratories	
ImageQuantTL 8.1	GE Healthcare Life Sciences	
Morpheus		https://software.broadinstitute.org/morpheus/
Fiji (ImageJ 1.51n)	Schindelin, Arganda-Carreras & Frise et al 2012	https://imagej.net/Fiji

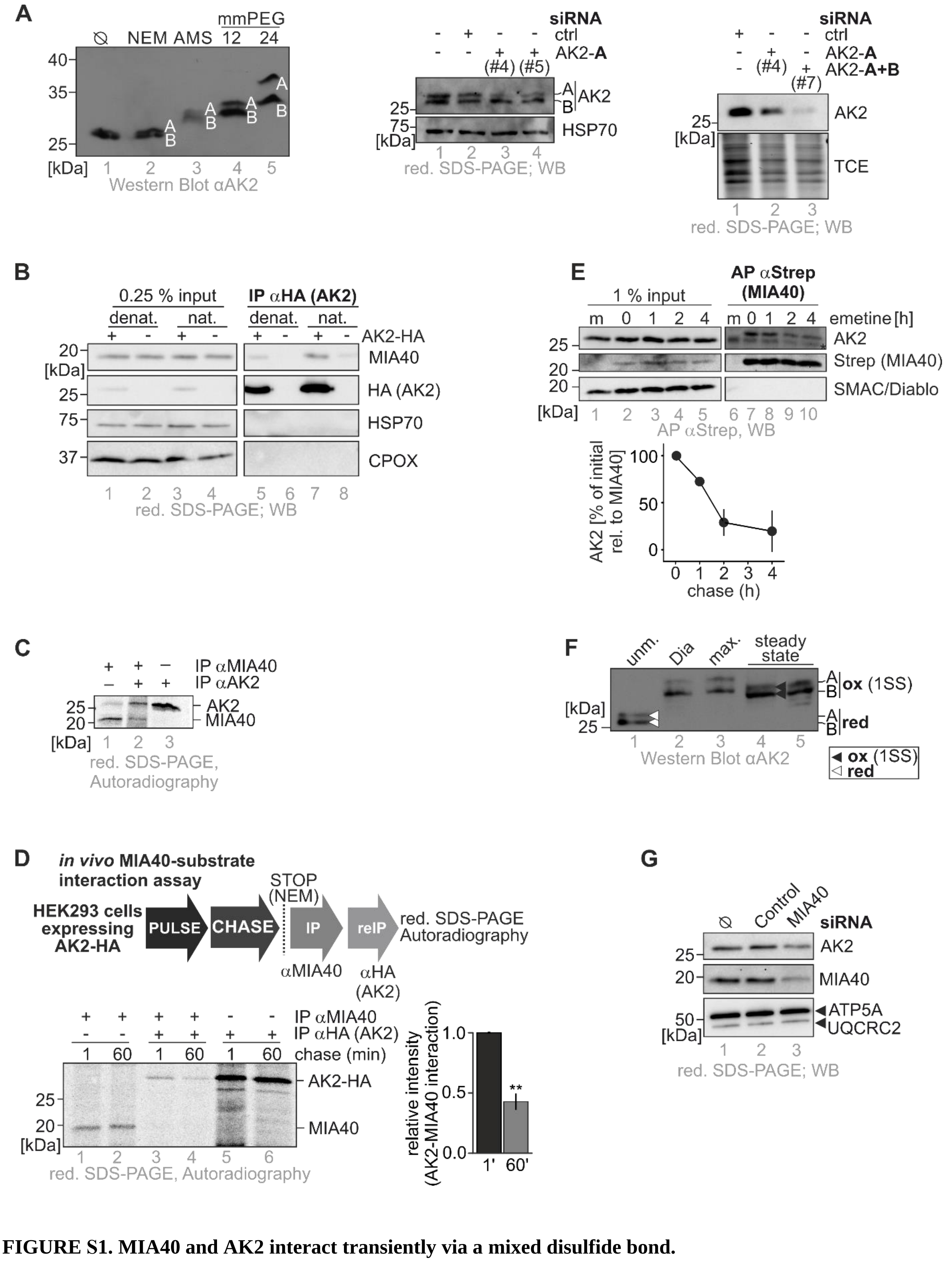

FIGURE S1. MIA40 and AK2 interact transiently via a mixed disulfide bond.

A) Assays to detect endogenous AK2 isoforms. AK2-A and AK2-B contain 4 and 3 cysteine residues, respectively. Using maleimide shift assays, both isoforms that otherwise have almost the same molecular weight can be distinguished (∅ represents the unmodified sample, other samples were modified with the indicated maleimide). HEK293 cells contain both, AK2-A and AK2-B (left panel). AK2-A and AK2-B differ in their mRNA and can thereby be detected. HEK293 cells were transfected with the indicated siRNAs directed either against isoform A of AK2 or against both AK2 isoforms. When we only targeted isoform A the upper band (AK2-A) became weaker (middle panel). When we treated cells with siRNA directed against both isoforms, the AK2

signal was almost completely lost (right panel). This assay again confirms that both AK2 isoforms are expressed in HEK293 cells. AMS, 4-acetamido-4′-maleimidylstilbene-2,2′-disulfonic acid; $mmPEG_{12}$ and $mmPEG_{24}$, polyethylene glycol-maleimide with 12 and 24 PEG units, respectively.

B) MIA40 and AK2 interact via a disulfide bond. Stable inducible cell lines that express AK2-HA were treated with N-ethyl maleimide (NEM) to stop thiol-disulfide exchange processes and lysed under native or denaturing conditions. Lysates were analysed by subsequent immunoprecipitations against HA (AK2- HA) and immunoblotting. Like classical MIA40 substrates, AK2 interacted with MIA40 also after denaturing lysis (Petrungaro, 2015). The IMS protein CPOX and the protein HSP70 served as negative controls that did not interact with AK2. 2% input was loaded as control for CPOX and 0.25% input was loaded for the other proteins.

C) Endogenous MIA40 and AK2 interact with each other. HEK293 cells were pulse-labelled for 10 min with 35S-methionine, were then treated with N-ethyl maleimide (NEM) to stop thiol-disulfide exchange processes and lysed under denaturing conditions. Lysates were analysed by subsequent immunoprecipitations (IP) against MIA40 followed by re-immunoprecipitation against AK2. As a control, AK2 and MIA40 were directly immunoprecipitated. Precipitates were analyzed by SDS-PAGE and autoradiography. The IP-re-IP approach recovered both MIA40 and AK2 (lane 2) indicating interaction of both proteins.

D) AK2 and MIA40 interact transiently during AK2 maturation. Cells stably expressing AK2-HA were pulse-labelled for 10 min with ^{35}S-methionine and then chased with cold methionine for the indicated times. NEM treatment and denaturing cell lysis were followed by immunoprecipitation against endogenous MIA40 and re-immunoprecipitation against HA. In such an assay, only newly synthesized proteins that after a certain chase period still interact with MIA40 would co-immunoprecipitate. Indeed, AK2 was lost from MIA40 over time indicating a transient interaction of both proteins (lanes 3 and 4). Reported values are the mean of 3 independent experiments; error bars represent ±SD. Student's t-test was performed. ** represents $p<0.01$.

E) AK2 and MIA40 interact transiently during AK2 maturation. Cells stably expressing MIA40-Strep were treated for the indicated times with the translation inhibitor emetine. Cells were lysed and native immunoprecipitations against MIA40-Strep were performed. Subsequently, precipitates were analysed by immunoblotting against MIA40 and AK2. Interaction between AK2 and MIA40 is lost over time indicating a transient interaction of both proteins. The IMS protein SMAC served as negative control that did not interact with MIA40. 1% input was loaded as control. m, mock sample treated with water. Reported values are the mean of 2 independent experiments; error bars represent ±SD.

F) Reproduction of AK2 redox state experiment. Experiment was performed as described in Figure 1C. Both isoforms of endogenous AK2 are partially oxidized.

G) siRNA-mediated depletion of MIA40 for the experiment presented in Figure 1E. 72 h before the experiment, cells were transfected with control siRNA or siRNA directed against MIA40 (∅ represents the untreated sample). Lysates were analysed by immunoblotting against the indicated proteins.

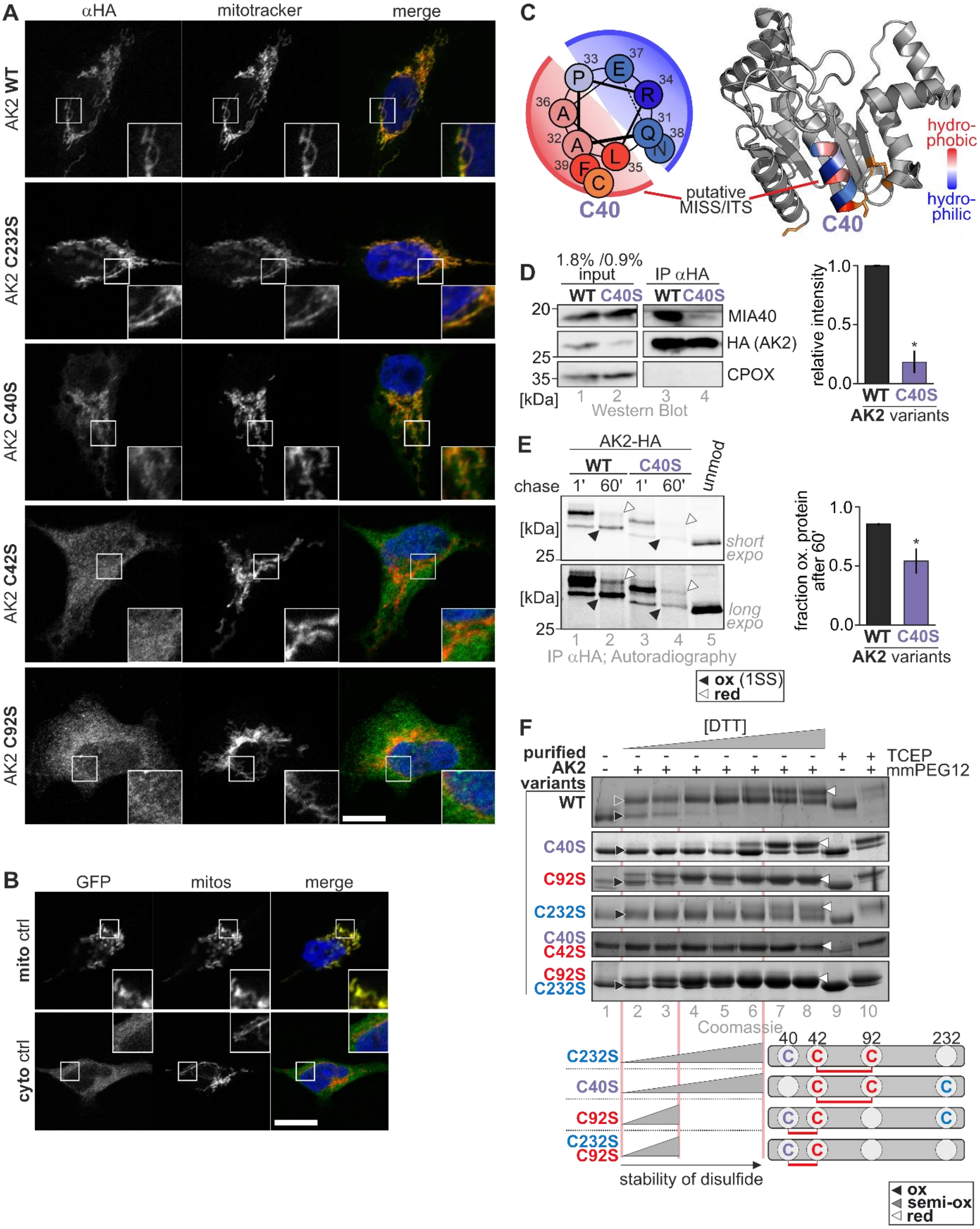

FIGURE S2. Cysteine 40 accelerates oxidative folding and mitochondrial import of AK2.

A,B) Representative immunofluorescence data for the localization of different AK2-HA constructs and the controls. GFP with a nuclear export sequence was used as cytosolic control while a Su9-GFP served as mitochondrial control. Experiment was performed as described in Figure 2C. Data shown in Figures 2C, 5B, S2 and S5 are derived from the same experiments. Data information: Scale bars: 10 µm.

C) Analysis of surrounding of AK2 cysteines reveals the presence of an amphipathic helix N-terminal to C40 (helical wheel representation) indicating a potential for initial interaction between MIA40 and C40 of AK2.

D) AK2 interacts with MIA40 via C40. Immunoprecipitation of $AK2^{WT}$ -HA and $AK2^{C40S}$ -HA to test for interaction with MIA40. Stable inducible cell lines expressing wild type (WT) or C40S variants of AK2- HA were treated with NEM and lysed under denaturing conditions. AK2-HA variants were precipitated using anti-HA-beads. Immunoblot analyses were performed against MIA40, HA and as control the IMS protein CPOX. Reported values are the mean of 2 independent experiments; error bars represent ±SD. Student's t-test was performed. * represents $p<0.05$. 1.8 and 0.9 % input were loaded as control for AK2WT and AK2C40S, respectively.

E) Oxidation of $AK2^{C40S}$ -HA is delayed compared to the wildtype. Oxidation kinetics experiment to test for maturation of $AK2^{C40S}$ -HA cysteine variants. Experiments were performed as described in Figure 1C, except HEK293 cells expressing AK2-HA cysteine variants and antibody directed against HA were used. Reported values are the mean of 3 independent experiments; error bars represent ±SD. Student's t-test was performed. * represents $p<0.05$.

F) A disulfide bond between C40 and C42 can in principle be formed and is unstable. Analysis of cysteine pattern in purified cysteine variants of AK2. Different cysteine variants of AK2 were heterologously expressed in E. coli and purified. Stability of their disulfide bonds was analysed by exposure to increasing amounts of DTT, subsequent TCA precipitation and mmPEG12 modification. The C42-C92 disulfide is more stable compared to the C40-C42 disulfide bond. This indicates that it is energetically possible to form the C42-C92 disulfide bond by rearrangement from the C40-C42 disulfide bond.

Supplemental Discussion to Figure S2

As far as we know, classical MIA40 substrate are oxidized directly to their native disulphide conformation. This is supported by their small size (<15 kDa) and a helix-loop-helix structure that positions cysteines belonging to a disulfide bond in the mature protein into close proximity. AK2 is a larger protein (ca 26 kDa) and has a very different more complex structure. Its three conserved cysteines are localized to the end of helices or β-sheets. For AK2, we thus propose a different folding mechanism that takes its structure into account. First, MIA40 interacts with C40 of AK2 driven by the MISS signal N-terminal to this cysteine. Then, a disulfide between C40 and C42 is formed that is instable. Thus, this non-native disulfide bond can easily be isomerized to the native C42-C92 disulfide bond. We propose that this disulfide shuffling mechanism ensures more rapid 'trapping' of AK2 in the IMS ensuring increased import efficiency. The AK2 import mechanism is not the only deviation in import of disulfide relay substrates; some substrates like MICU1 and TIMM17 contain an N-terminal MTS that guides them to the IMS independently of the disulfide relay. Others employ additional targeting information, e.g. CHCHD3/MIC19 an N-terminal myristoylation that supports import.

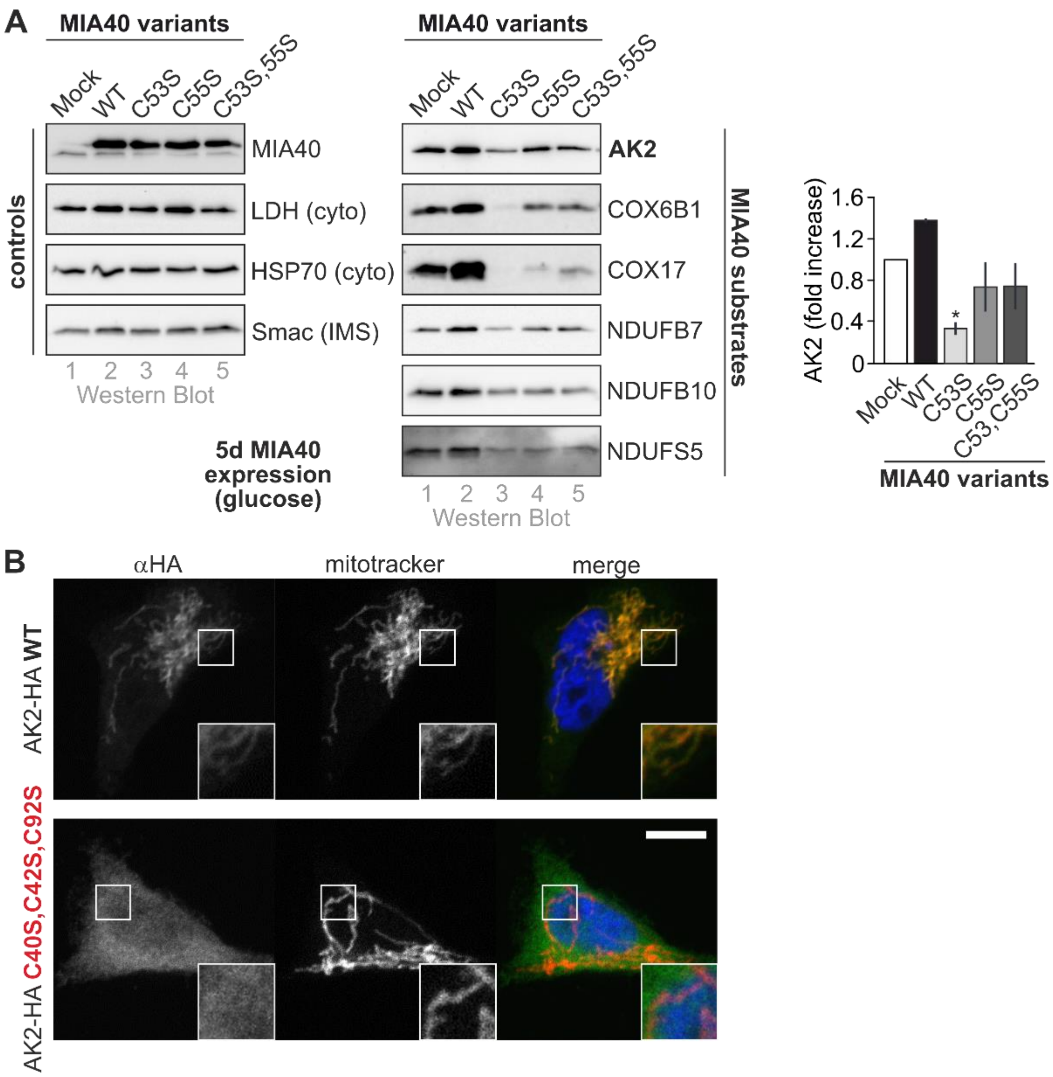

FIGURE S3. Expression of dominant MIA40 variants results in AK2 loss.

A) Expression of dominant-negative MIA40 variants decreases AK2 levels. We recently established that different cysteine mutants of MIA40 exhibit dominant negative effects on cellular levels of MIA40 substrates. While their overexpression results in decreased substrate levels, overexpression of wild type MIA40 leads to increased substrate levels (Habich et al, 2019). Expression of MIA40 variants from stable inducible cells was induced for 5 days in glucose-containing medium. Cells were lysed and protein levels were analysed by SDS-PAGE and immunoblot against the indicated proteins. Reported values are the mean of 3 independent experiments; error bars represent ±SD. Student's t-test was performed. *represents $p<0.05$.

B) AK2^{C40S,C42S,C92S} localizes to the cytosol. Experiment was performed as described in Figure 2C. Data information: Scale bars: 10 μm.

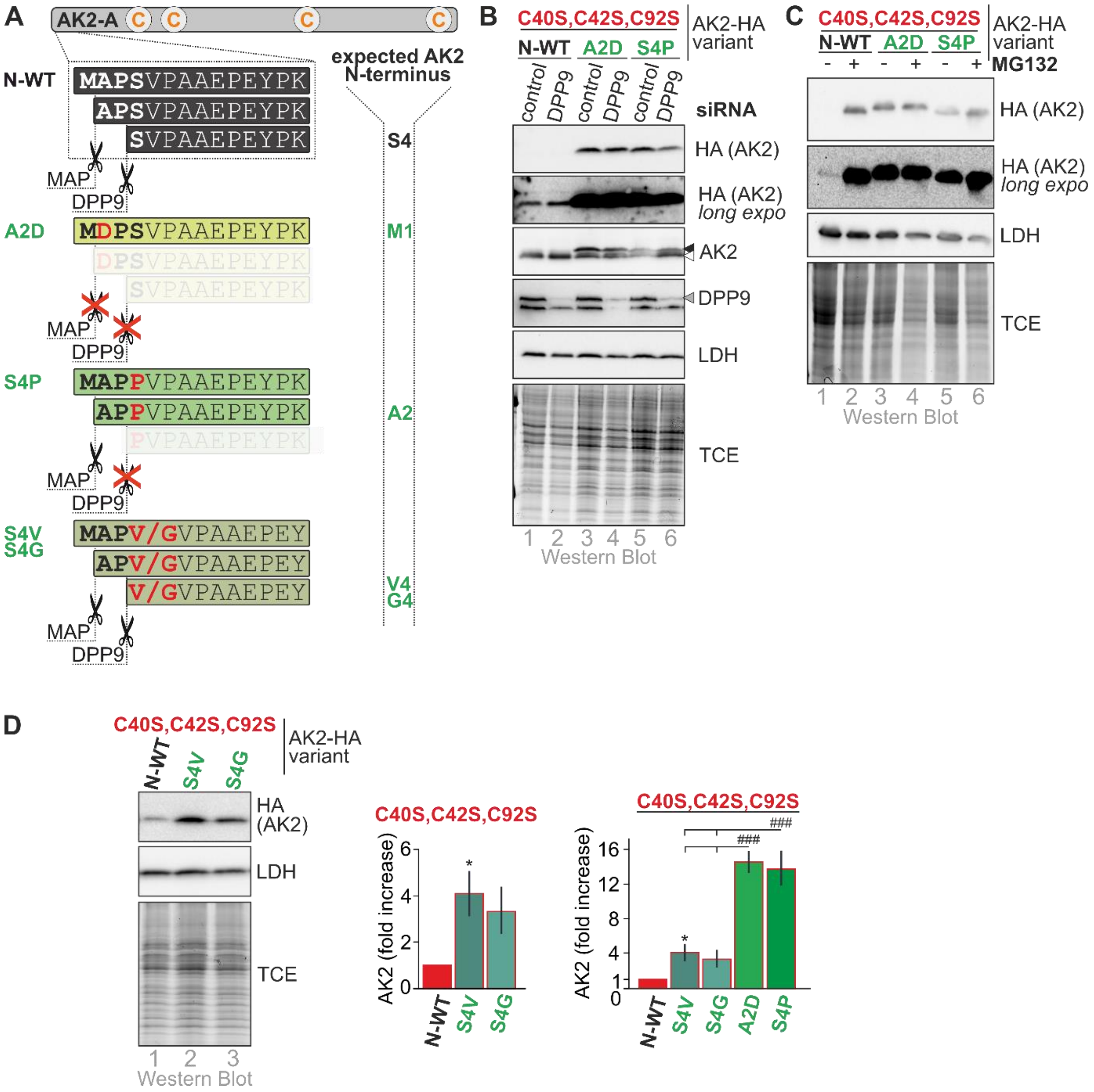

FIGURE S4. DPP9-insensitive AK2-HA variants are not influenced by DPP9 depletion or inhibition and only in part by proteasomal inhibition.

A) Design of DPP8/9-insensitive AK2 mutants. The AK2 mutations A2D and S4P should prevent MAP and DPP cleavage, respectively and thus stabilize AK2. S4V and S4G mutations should lead to different N-termini of AK2 after DPP9 cleavage.

B,C) AK2-HA variants were subjected to siRNA-mediated depletion of DPP9 (B , N=1) or to the proteasomal inhibitor MG132 (C , N=1). White and black arrow heads indicate the endogenous and HA-tagged AK2, respectively. The gray arrow head indicates the DPP9 band. In all cases, the DPP9-insensitive variants of AK2-HA were not affected by the treatment, while AK2-HA variants with an unchanged N-terminus were.

D) Changing the mature N-terminus of AK2 to valine (S4V) or glycine (S4G) instead of serine increased levels of AK2-HA although not to the same extent as completely preventing DPP9 cleavage (S4P). Experiment was done as in Figure 4F. Reported values are the mean of 4 independent experiments; error bars represent ±SD. ANOVA/Tukey's post hoc tests was performed. * represents $p<0.05$ and ### represents $p<0.001$.

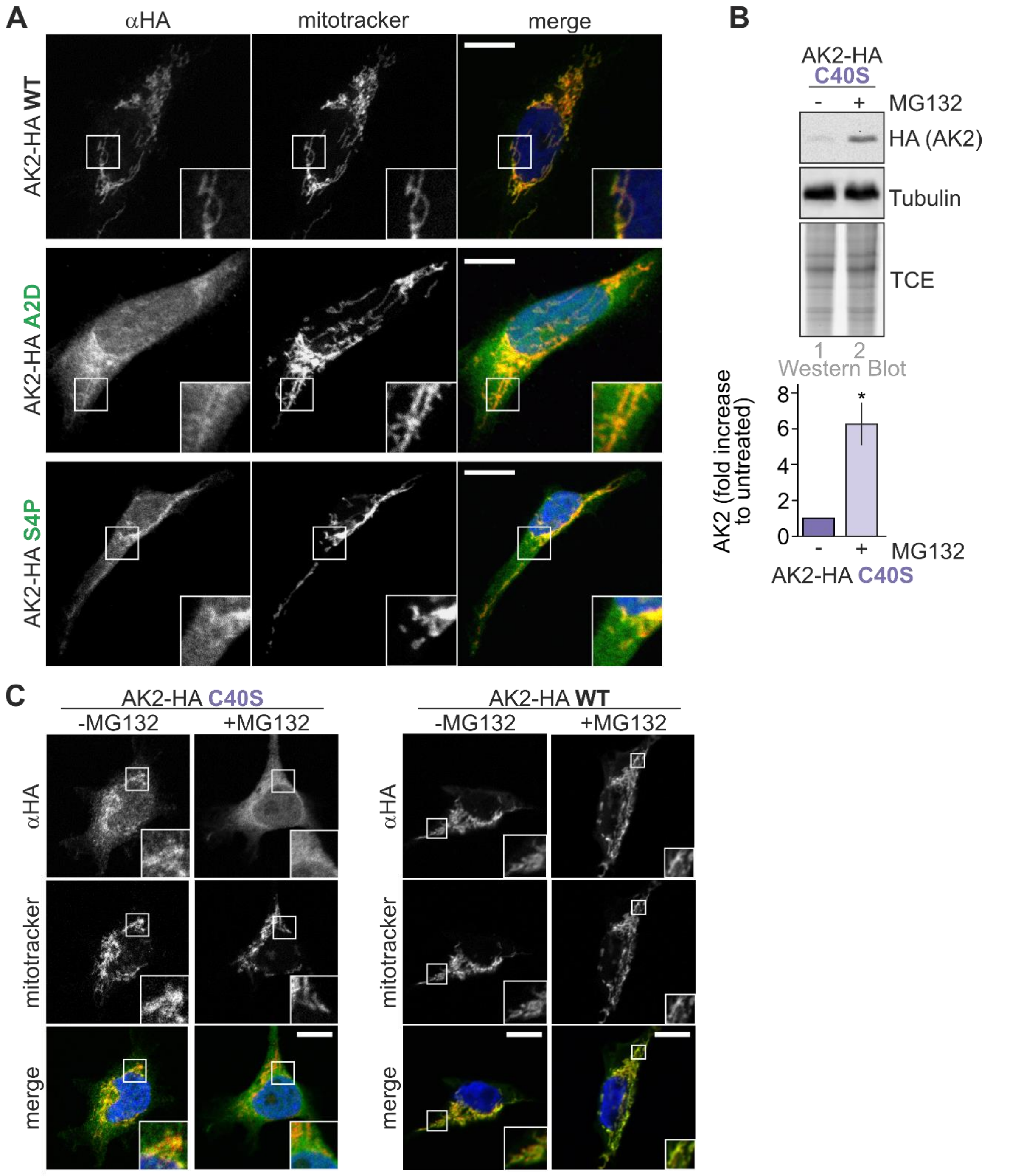

FIGURE S5. Preventing DPP9 activity results in cytosolic accumulation of AK2

A) Representative immunofluorescence data for the localization of DPP9-insensitive AK2-HA constructs (Figure 5B). Data information: Scale bars: 10 µm.

B) AK2^{C40S} can be stabilized by proteasomal inhibition. Experiment was performed as described in Figure 3D. Reported values are the mean of 3 independent experiments; error bars represent ±SD. Student's t-test was performed. * represents $p<0.05$.

C) Representative immunofluorescence data for the localization of DPP9-insensitive AK2-HA constructs (Figure 5C). Cytosolic localization of AK2^{C40S} but not AK2 wildtype increases upon proteasomal inhibition. Data information: Scale bars: 10 µm.

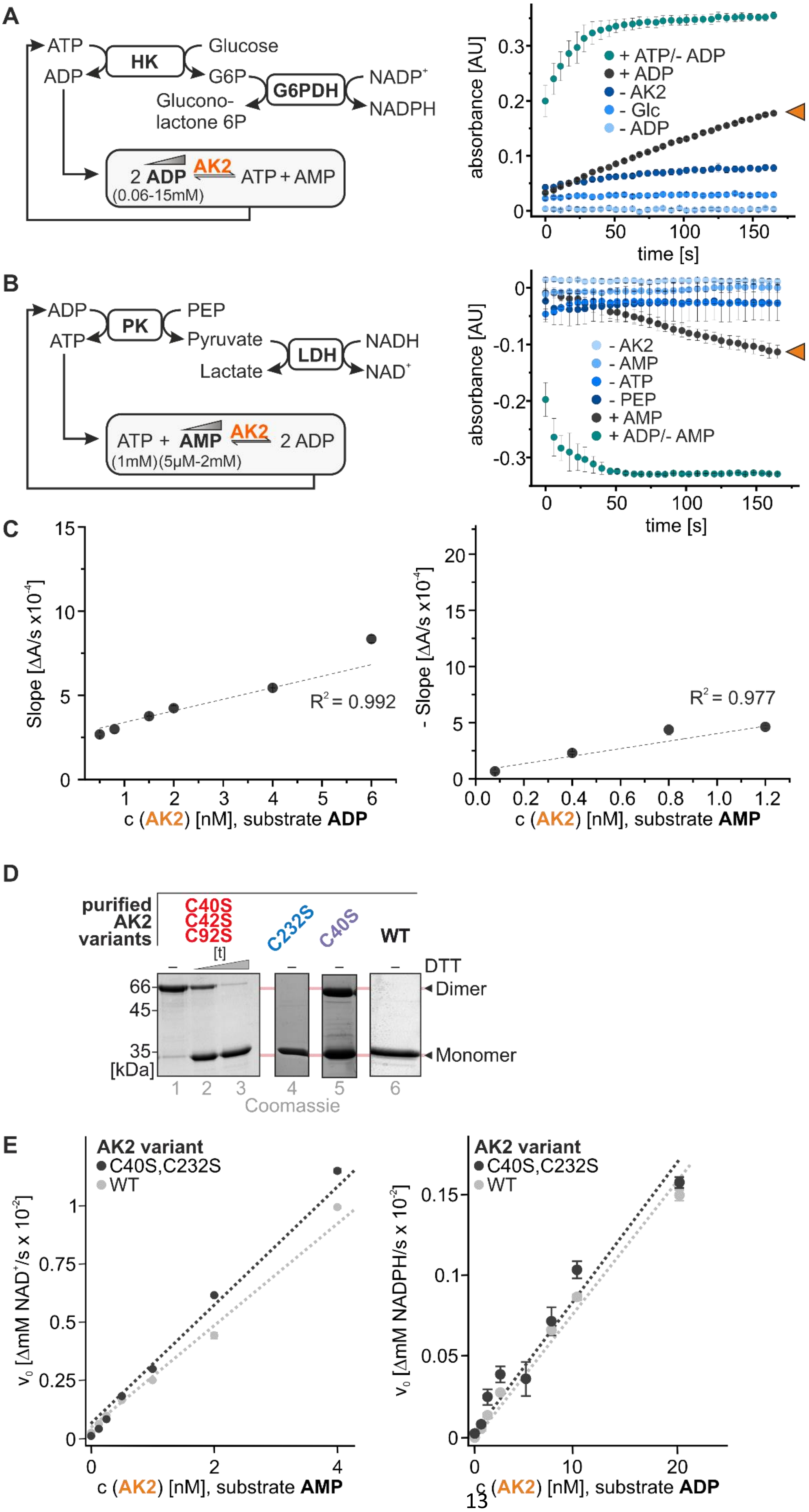
A
ATP
ADP
HK
Glucose
G6P
G6PDH
NADP+
NADPH
Glucono-
lactone 6P
2 ADP
AK2
ATP + AMP
(0.06-15mM)
absorbance [AU]
time [s]
+ ATP/- ADP
+ ADP
- AK2
- Glc
- ADP
B
ADP
ATP
PK
PEP
Pyruvate
LDH
NADH
NAD+
Lactate
ATP + AMP
AK2
2 ADP
(1mM)(5μM-2mM)
absorbance [AU]
time [s]
- AK2
- AMP
- ATP
- PEP
+ AMP
+ ADP/- AMP
C
Slope [ΔA/s x10-4]
R2 = 0.992
c (AK2) [nM], substrate ADP
- Slope [ΔA/s x10-4]
R2 = 0.977
c (AK2) [nM], substrate AMP
D
purified
AK2
variants
C40S
C42S
C92S
C232S
C40S
WT
[t]
DTT
66
45
35
[kDa]
Dimer
Monomer
1 2 3 4 5 6
Coomassie
E
AK2 variant
C40S,C232S
WT
v0 [ΔmM NAD+/s x 10-2]
c (AK2) [nM], substrate AMP
AK2 variant
C40S,C232S
WT
v0 [ΔmM NADPH/s x 10-2]
c (AK2) [nM], substrate ADP

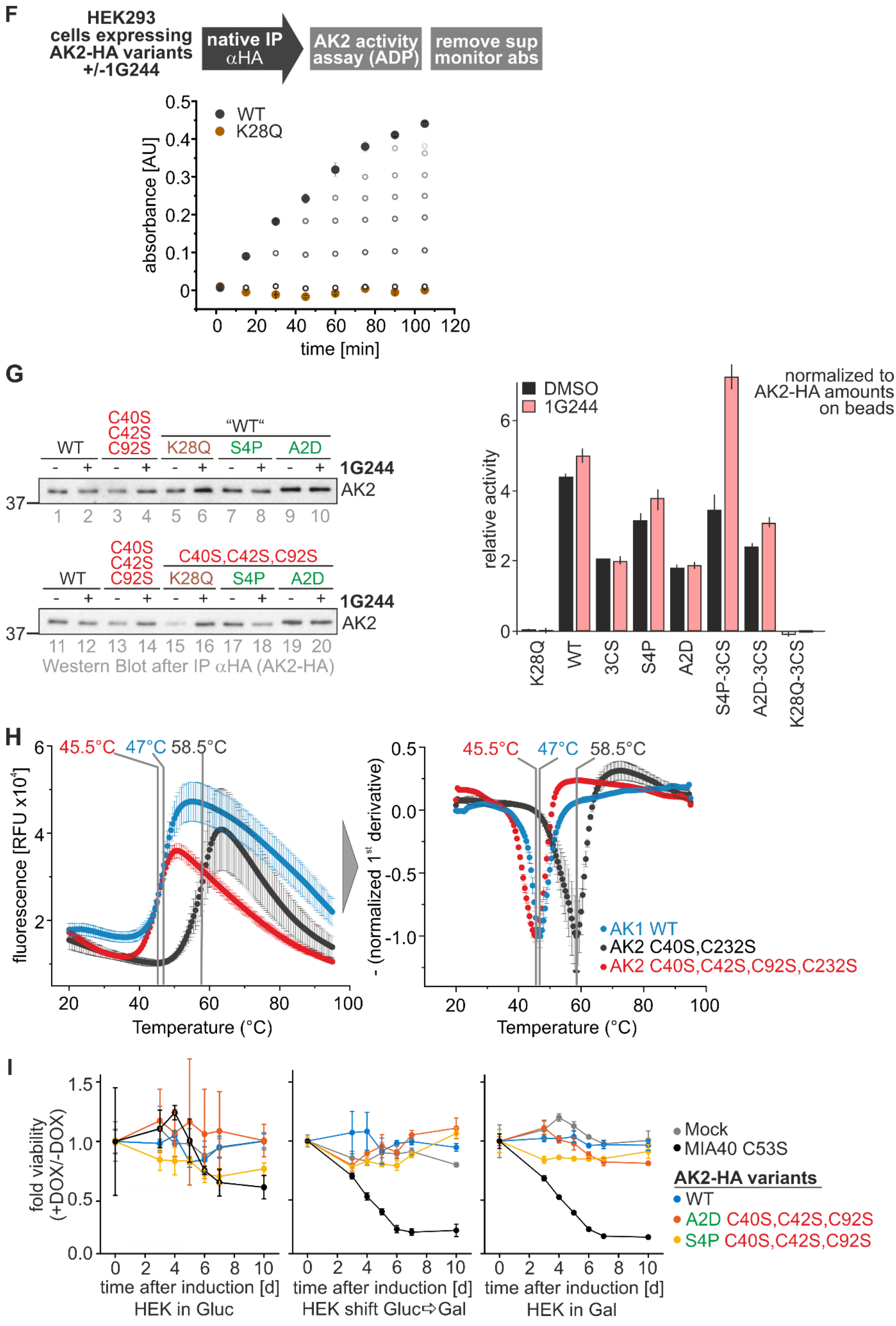

F
HEK293 cells expressing AK2-HA variants +/-1G244
native IP αHA
AK2 activity assay (ADP)
remove sup monitor abs
WT
K28Q
absorbance [AU]
time [min]
G
WT
C40S C42S C92S
"WT"
K28Q
S4P
A2D
1G244
AK2
37
C40S,C42S,C92S
Western Blot after IP αHA (AK2-HA)
DMSO
1G244
normalized to AK2-HA amounts on beads
relative activity
K28Q
WT
3CS
S4P
A2D
S4P-3CS
A2D-3CS
K28Q-3CS
H
45.5°C
47°C
58.5°C
fluorescence [RFU x10^4]
Temperature (°C)
- (normalized 1st derivative)
AK1 WT
AK2 C40S,C232S
AK2 C40S,C42S,C92S,C232S
I
fold viability (+DOX/-DOX)
time after induction [d]
HEK in Gluc
HEK shift Gluc⇨Gal
HEK in Gal
Mock
MIA40 C53S
AK2-HA variants
WT
A2D C40S,C42S,C92S
S4P C40S,C42S,C92S

FIGURE S6. Control experiments for AK2 activity assays, stability of AK2 variants, and impact of AK2 variants on cellular proliferation.

A,B) Control experiments for activity assays. The activity assays towards the substrates ADP (A) and AMP (B) were performed in the absence of individual components of the assay solution or with the respective positive controls ATP (A) and ADP (B). Only the positive controls and the full set of assay components allows for the in vitro assay to monitor AK2 activity with respect to the indicated substrates. Orange arrow head indicates the reaction mix containing all constituents of the "real" assay. Reported values are the mean of 3 independent experiments; error bars represent ±SD.

C) Linearity of AK2 assays (substrates ADP or AMP) with respect to AK2 amounts. In the assay, the amounts of AK2 were varied. In the indicated range observed turn over kinetics correlate in a linear fashion with AK2 amounts.

D) Analysis of heterologously expressed and purified AK2 variants. AK2 variants were expressed in E. coli and purified. Purified $AK2^{WT}$ showed a band migrating at the height of an AK2 monomer, while all cysteine mutant variants still containing C232 migrated in part as dimer on a Coomassie-stained gel. The dimer band disappeared upon treatment with DTT, and it was also not present in $AK2^{C232S}$ indicating that purified AK2 formed these unphysiological dimers via a disulfide bond involving C232.

E) Comparison of activity of the two AK2 variants $AK2^{WT}$ and $AK2^{C40S,C232S}$ in dependence of their concentration towards their substrates ADP and AMP. In the assay, the amounts of AK2 were varied. In the indicated range, observed turn over kinetics correlate in a linear fashion with AK2 amounts. Both AK2 variants exhibited very similar profiles. Reported values are the mean of 3 independent experiments; error bars represent ±SD.

F) Activity assay of AK2 isolated from HEK293 cells. Control experiment: The assay towards the substrate ADP was performed after isolation of AK2 variants from HEK293 cells. AK2 still bound to HA-beads were incubated for the indicated times with an assay buffer containing all components and after the indicated times, some supernatant was removed for assessment by UV/Vis spectroscopy (filled circles). These samples were after different times repeatedly measured (hollow circles with different gray coloring) to test whether the reaction progressed in the absence of isolated AK2. Only in the presence of HA-beads carrying functional AK2 (WT but not K28Q) did the reaction progress. Reported values are the mean of 3 independent experiments; error bars represent ±SD.

G) Amounts of proteins immunoprecipitated for the activity assay in Figure 6E. AK2 variants were isolated from HEK293 cells stably expressing these variants by immunoprecipitation and subjected to the activity assay depicted in Figure 6E. Activity was thereby assessed with AK2 still bound to antibody-beads. Afterwards, beads were boiled to release AK2, and the lysates were analysed by immunoblotting against AK2. Protein levels were quantified and used to normalize AK2 activities depicted in Figure 6E. Although this kind of assay only provides an estimate of precipitated AK2, there is a clear trend indicating that specific activity of AK2 variants might be similar. Reported values are the mean of 3 independent experiments; error bars represent ±SD.

H) Thermofluor assay to assess the thermal stability of AK2 variants. In the thermofluor assay, a dye binds to hydrophobic areas in a protein and thereby begins to fluoresce. left: Raw data. The fluorescence in RFU x 104 was plotted against the temperature for AK1 (blue curve), $AK2^{C40S,C232S}$ (black) and $AK2^{C40S,C42S,C92S,C232S}$ (red). right: Normalized negative first derivative of the raw data. The normalized first derivative in RFU/°C was plotted against the temperature for AK1 (blue curve), $AK2^{C40S,C232S}$ (black) and $AK2^{C40S,C42S,C92S,C232S}$ (red). Data was normalized by each minimum value in RFU/°C. The inflection point of the curve represents the melting temperature of the protein. The estimated melting points are marked by a grey line. Reported values are the mean of 3 independent experiments; error bars represent ±SD.

I) Proliferation of cells upon overexpression of AK2 variants. Expression of AK2 variants was induced for up to 10 days in glucose or galactose-containing medium. Cells were analyzed by PrestoBlue cell viability reagent. Fold viability is presented as the viability data of induced cells divided by the data of non-induced cells of the same cell line. The dominant MIA40 variant, $MIA40^{C53S}$ served as negative control. A value of 1 indicates that cells with and without protein induction grow equally well. Expression of cytosolic AK2 did not result in a decrease of cellular viability. Reported values are the mean of 3 independent experiments; error bars represent ±SD.

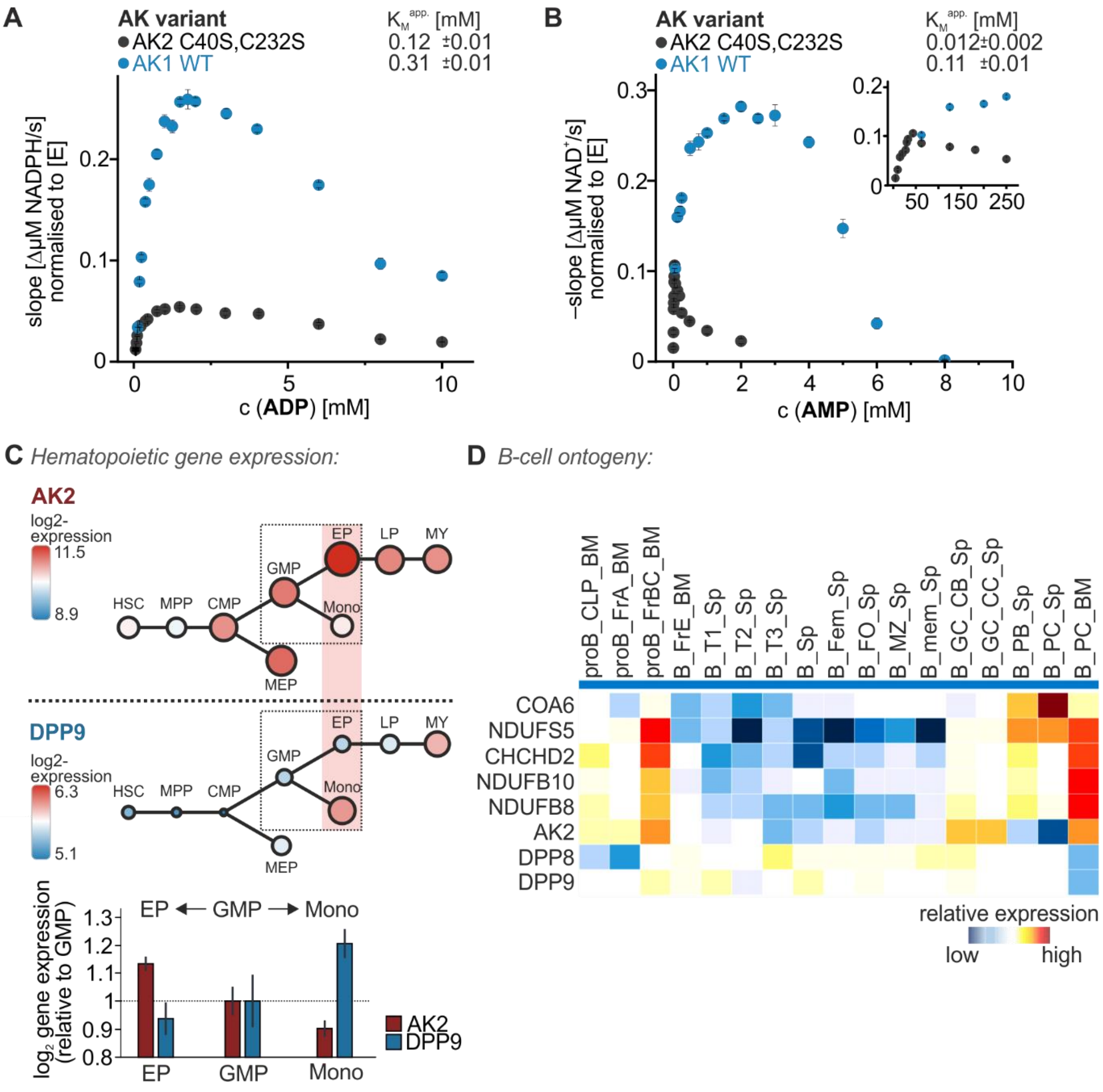

FIGURE S7. AK2 expression patterns and AK1-AK2 activity comparison

A,B) Comparison of AK1 and AK2 activity. Experiments were performed as described in Figure 6. The curves for AK2 depicted in A and B are the curves also shown in Figure 6. AK2 has a higher specific activity compared to AK1, and towards AMP as a substrate, AK1 also shows a less pronounced substrate inhibition behavior. Reported values are the mean of 3 independent experiments; error bars represent ±SD.

C) Hematopoietic gene expression data for AK2 and DPP9. Gene expression data during hematopoiesis was retrieved from BloodSpot (http://servers.binf.ku.dk/bloodspot). Color and size of branching points indicates the expression level. Data sets: AK2: 208967_s_at, DPP9: 226916_x_at. HSC, hematopietic stem cell; MPP, multipotential progenitors; CMP, common myeloid progenitor; MEP, megakaryocyte/erythroid progenitor; GMP, granulocyte/monocyte progenitor; Mono, monocytes; EP, early promyelocyte; LP, late promyelocyte; MY, myelocyte. DPP9 expression is maintained at low levels during differentiation into early promyelocytes but increase upon differentiation into monocytes. AK2 expression exhibits the inverse pattern. Reported values are the mean of 3-6 independent experiments as reported on the BloodSpot database; error bars represent ±SD.

D) Hierarchical clustering of gene expression of Dpp8/9 and the identified Dpp8/9 substrates (Figure 7) during B cell ontogeny. RNAseq data from purified B cell subsets publicly available in the Immgen database (Heng et al., Nat. Comm., 2008; www.immgen.org) were used for hierchartical clustering using 17 the MyGeneset tool (http://rstats.immgen.org/MyGeneSet_New/index.html). Sorted B cells were as follows, with original sort protocols and analyses available on http://rstats.immgen.org/Skyline/skyline.html: proB_common lymphoid progenitor (CLP)_bone marrow (BM): CD93+, CD117+, IL7Ra+, CD45R-, proB_Hardy Fraction (Fr) A_BM: CD93+, CD117+, IL7Ra+, CD45R-, preB_FrBC_BM: CD19+, CD43+, heat stable antigen (HSA)+, B_FrE_BM:

CD19+, Immunoglobulin (Ig)M+, CD19+, CD43-, HSA+, B_FrE_BM: CD19+, IgM+, CD19+, CD43-, HSA+, B_transitional (T)1_Spleen (Sp): CD19+, IgM++, CD45R+, CD93+, CD23-, B_T2_Sp: CD19+, IgM++, CD45R+, CD93+, CD23+, B_T3_Sp: CD19+, IgM+, CD45R+, CD93+, CD23+, B_Sp: CD19+, IgM+, T cell receptor β-, B_Female (Fem)_Sp: CD19+, IgM+, T cell receptor β-, B_Follicular (FO)_Sp: CD19+, IgM+, CD45R+, CD93-, CD23+, CD43-, CD5-, B_marginal zone (MZ)_Sp: CD19+, IgM++, CD45R+, CD93-, CD23-, CD21/35++, CD43-, CD5-, B_memory (mem)_Sp: CD19+, B220+, IgG+, IgD-, Fas-, CD38+, B_germinal center (GC)_centroblasts (CB)_Sp: CD19+, B220+, IgD-, Fas+, CD38-, Cxcr4high, CD86low, B_GC_centrocytes (CC)_Sp: CD19+, B220+, IgD-, Fas+, CD38-, Cxcr4low, CD86high, B_plasmablasts (PB)_Sp: Blimp1-GFPlow, CD138+, NK1.1-, CD11b-, TCRb-, MHCII++, B_plasma cells (PC)_Sp: Blimp1-GFPhigh, CD138+, NK1.1-, CD11b-, TCRb-, MHCIIlow, B_PC_BM: Blimp1-GFPhigh, CD138+, NK1.1-, CD11b-, TCRb-, MHCIIlow. Hardy fractions refer to the originial identification of the respective subsets with the described markers by R. Hardy et al. (Hardy, RR et al., J. Exp. Medicine, 1991). Analysis of gene expression of DPP8/9 and its substrates during B cell ontogeny reveals high expression of DPP8/9 substrates in B cell stages that are metabolically most active (pro/pre B cells, Urbanzcyk et al., 2018; germinal center cells and plasma cells, Lam & Bhattarachaya, 2018) and a strikingly inverse correlation of DPP8/9 expression and their substrates in bone marrow plasma cells.
