# Supplementary figures and images for: Proteasomal degradation induced by DPP9‐mediated processing competes with mitochondrial protein import
Source: EMBO J. 2020 Aug 20;39(19):e103889. doi: 10.15252/embj.2019103889 (PMC7527813; doi:10.15252/embj.2019103889)

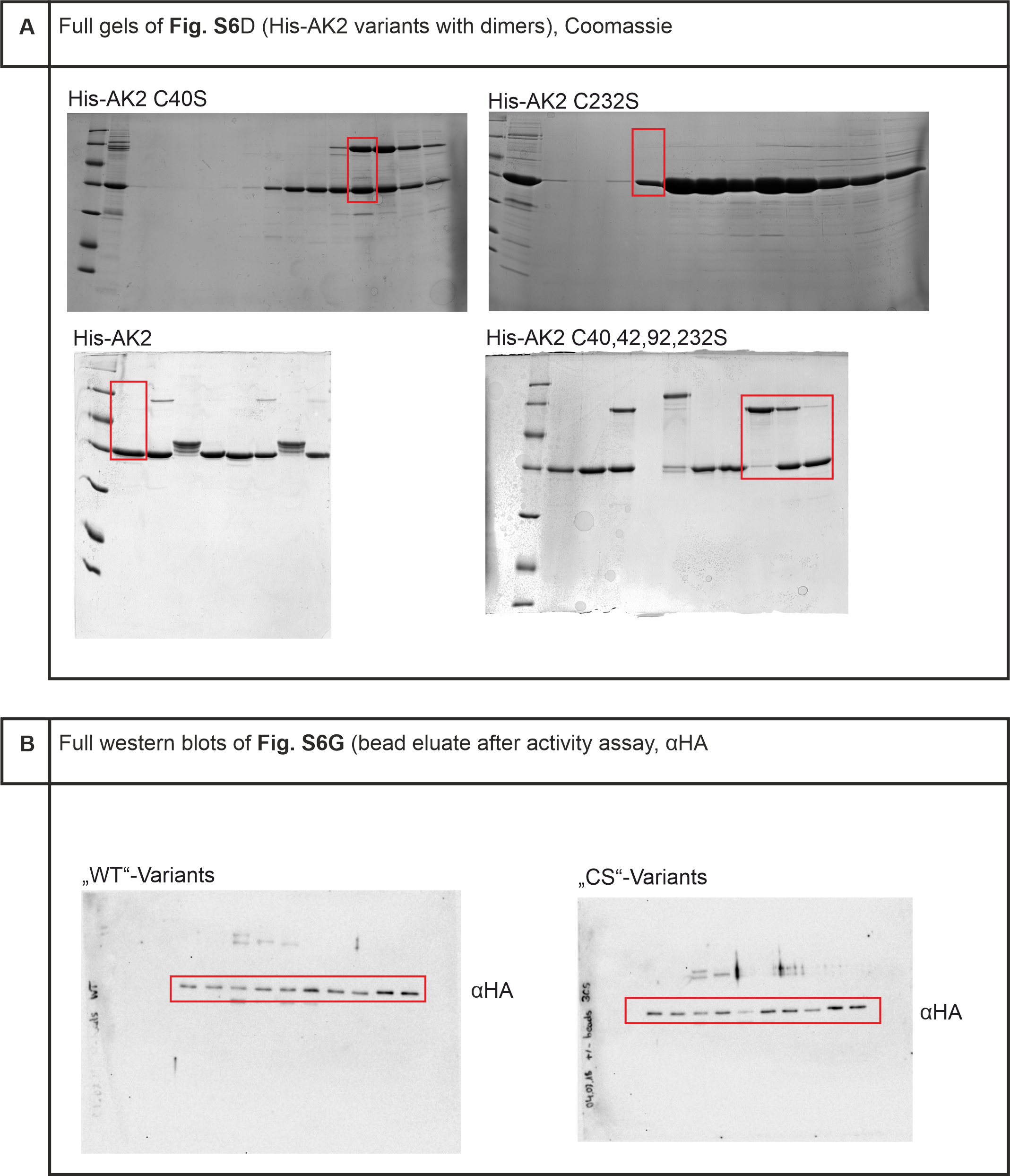

Supplement: Supplementary file 3 — Source Data for Appendix [file EMBJ-39-e103889-s010.zip › Appendix_Figure_Source_Data/S6-a.png]

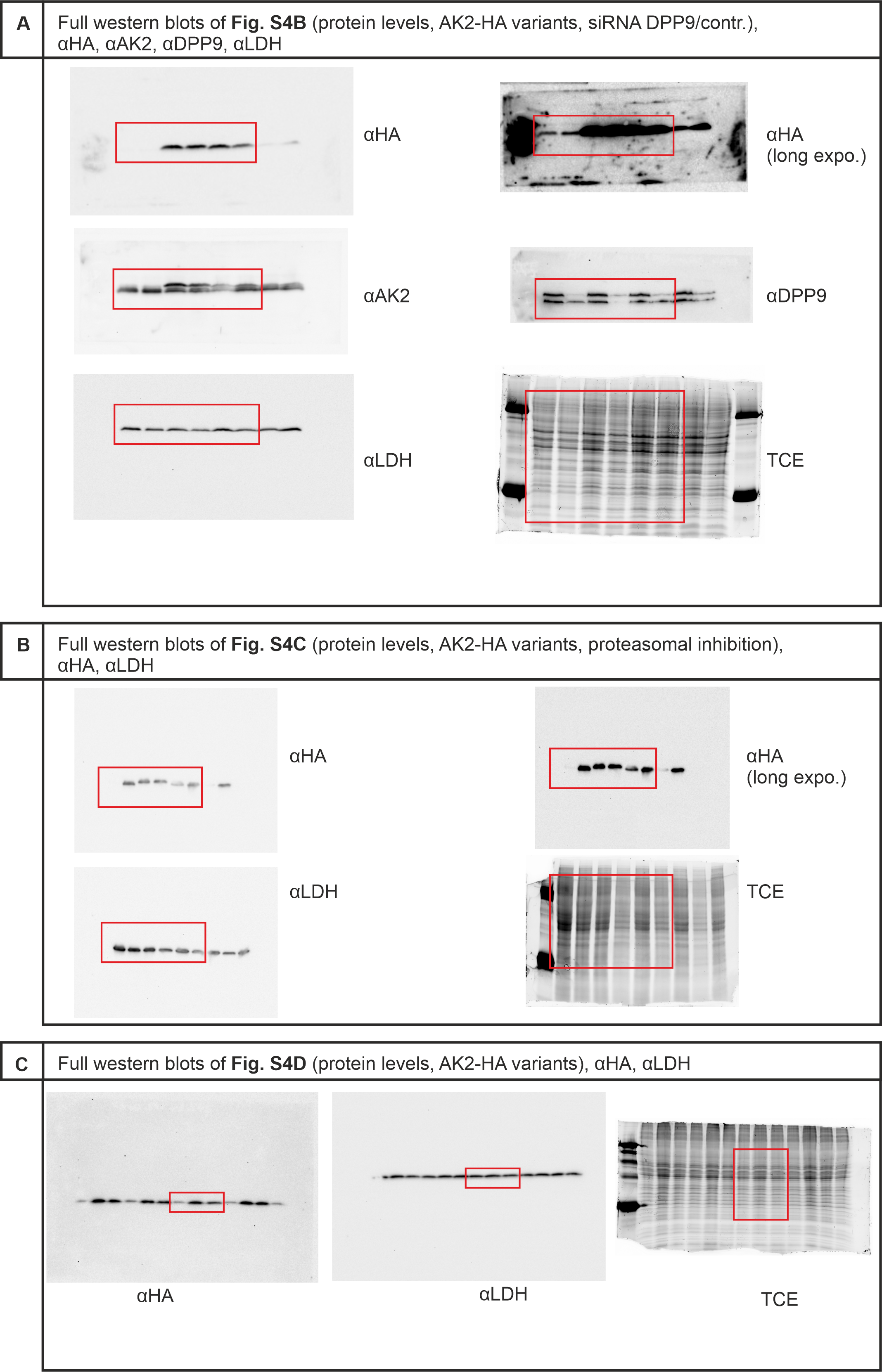

Supplement: Supplementary file 3 — Source Data for Appendix [file EMBJ-39-e103889-s010.zip › Appendix_Figure_Source_Data/S4-a.png]

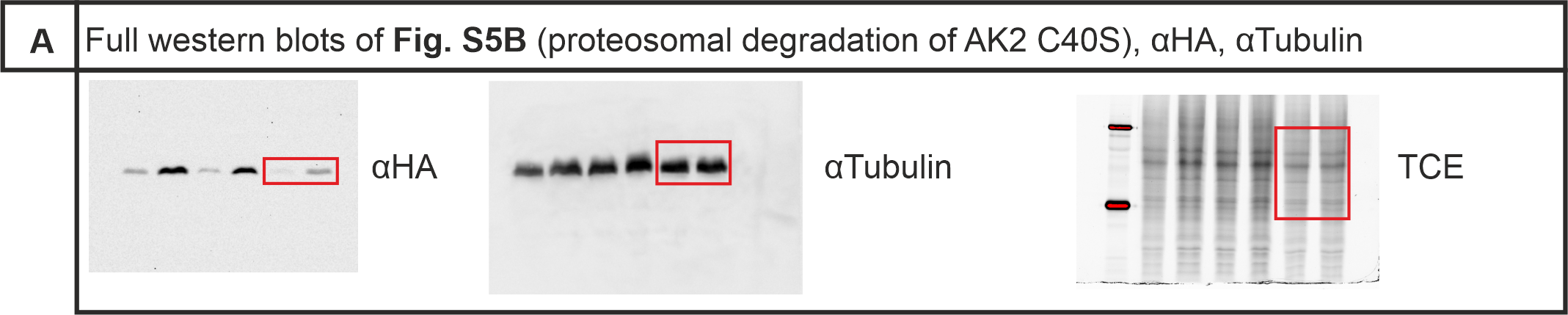

Supplement: Supplementary file 3 — Source Data for Appendix [file EMBJ-39-e103889-s010.zip › Appendix_Figure_Source_Data/S5-a.png]

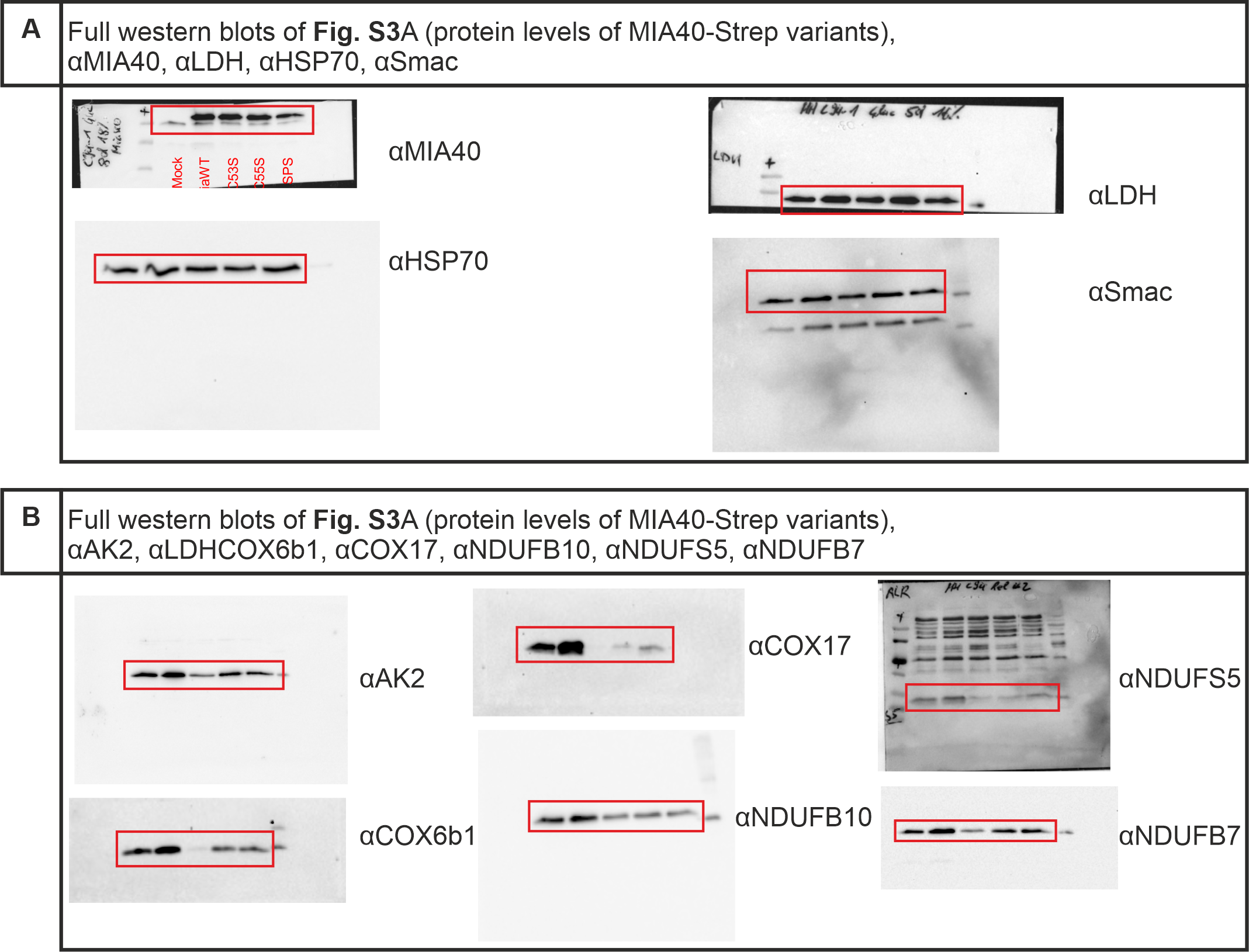

Supplement: Supplementary file 3 — Source Data for Appendix [file EMBJ-39-e103889-s010.zip › Appendix_Figure_Source_Data/Appendix_FigS3_Source_Data/S3-a.png]

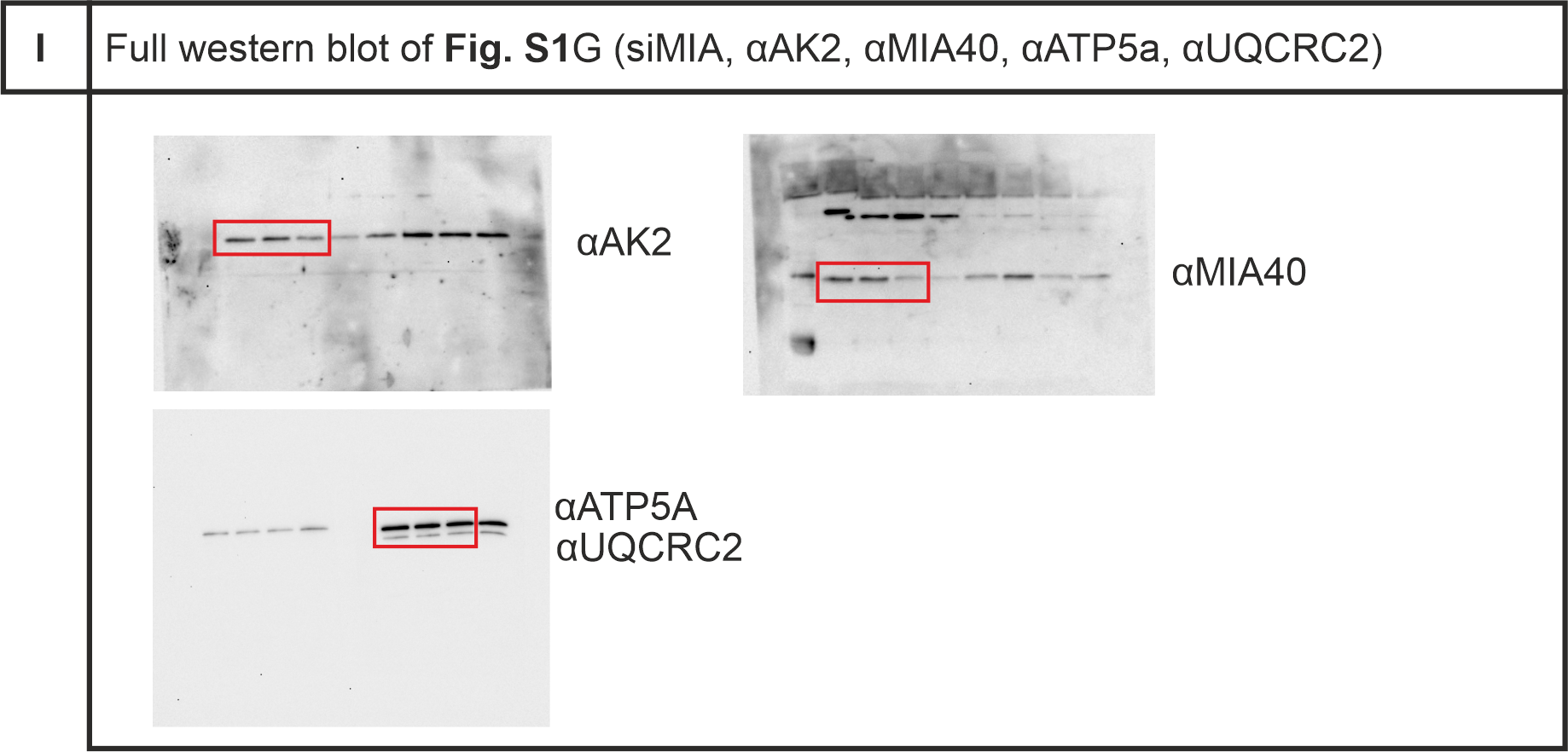

Supplement: Supplementary file 3 — Source Data for Appendix [file EMBJ-39-e103889-s010.zip › Appendix_Figure_Source_Data/Appendix_FigS1_Source_Data/S1-c.png]

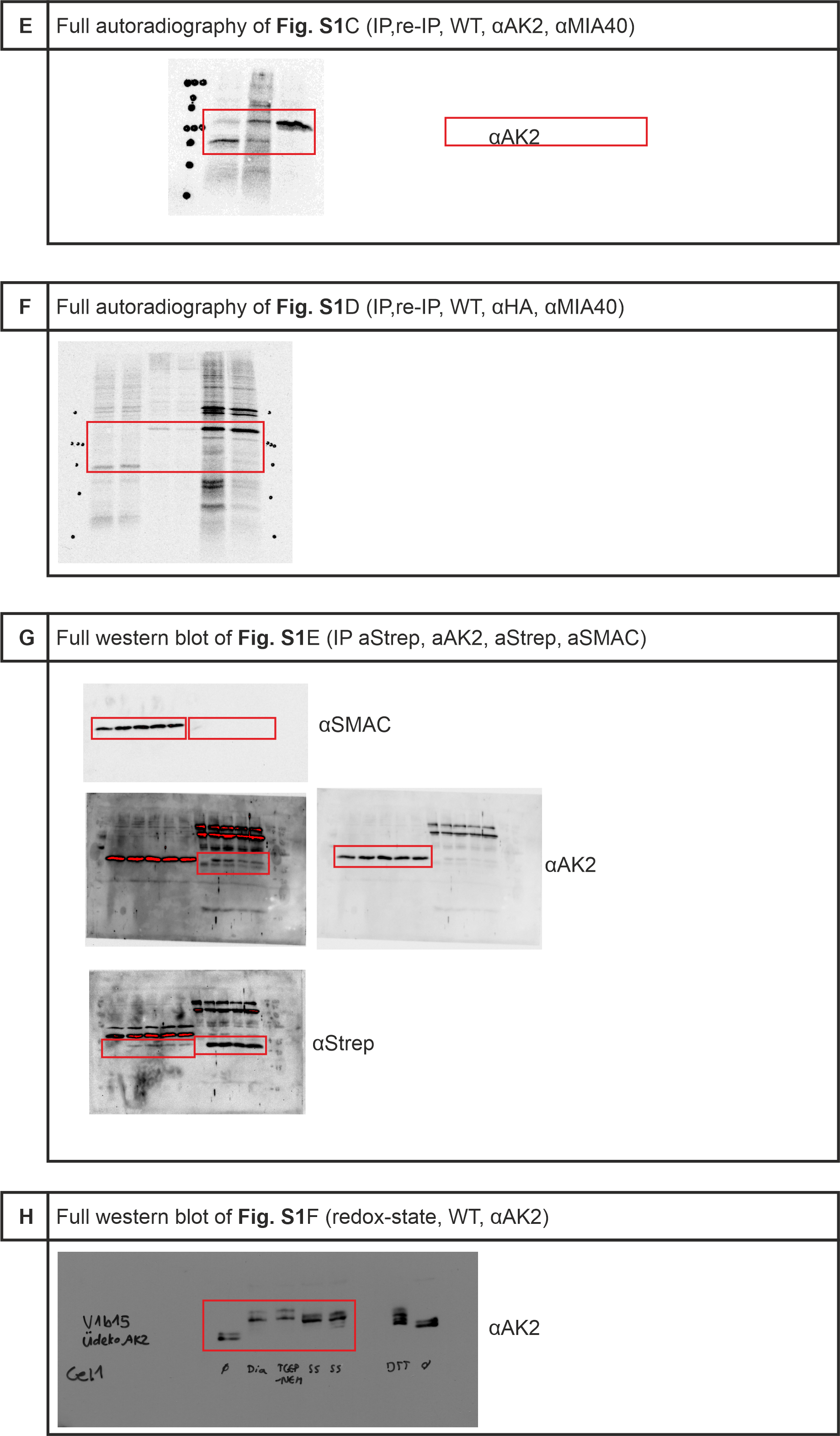

Supplement: Supplementary file 3 — Source Data for Appendix [file EMBJ-39-e103889-s010.zip › Appendix_Figure_Source_Data/Appendix_FigS1_Source_Data/S1-b.png]

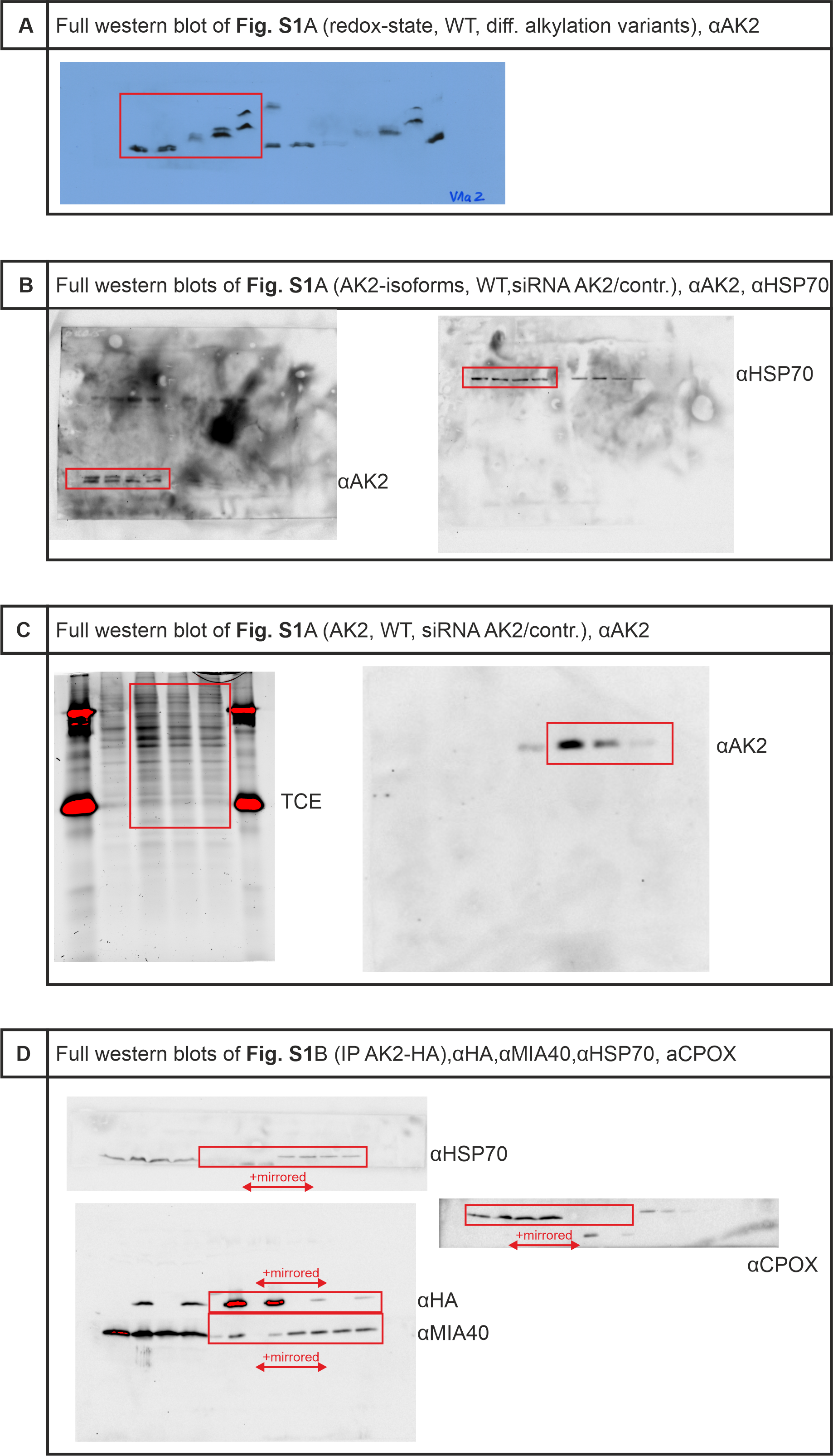

Supplement: Supplementary file 3 — Source Data for Appendix [file EMBJ-39-e103889-s010.zip › Appendix_Figure_Source_Data/Appendix_FigS1_Source_Data/S1-a.png]

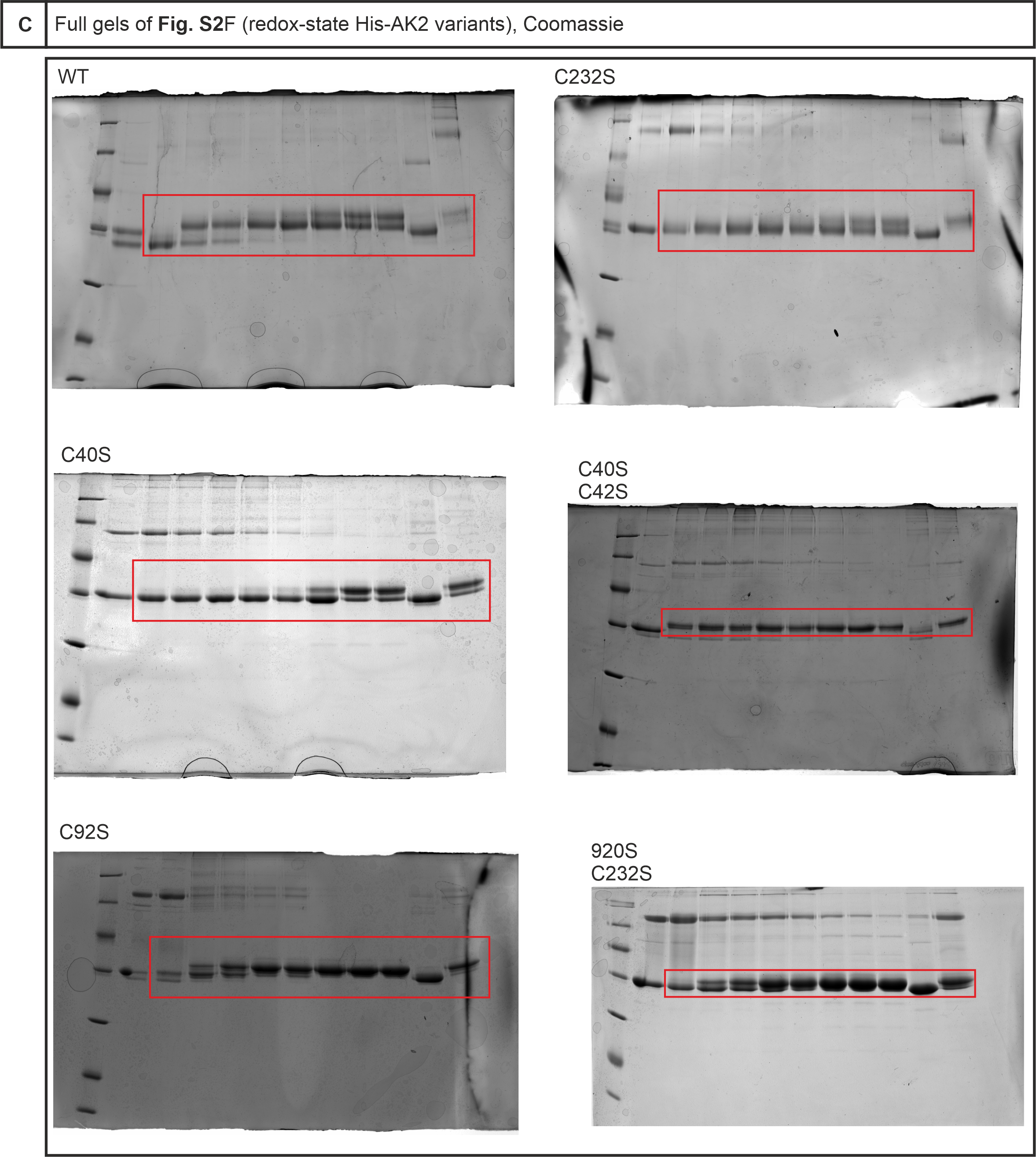

Supplement: Supplementary file 3 — Source Data for Appendix [file EMBJ-39-e103889-s010.zip › Appendix_Figure_Source_Data/Appendix_FigS2_Source_Data/S2-b.png]

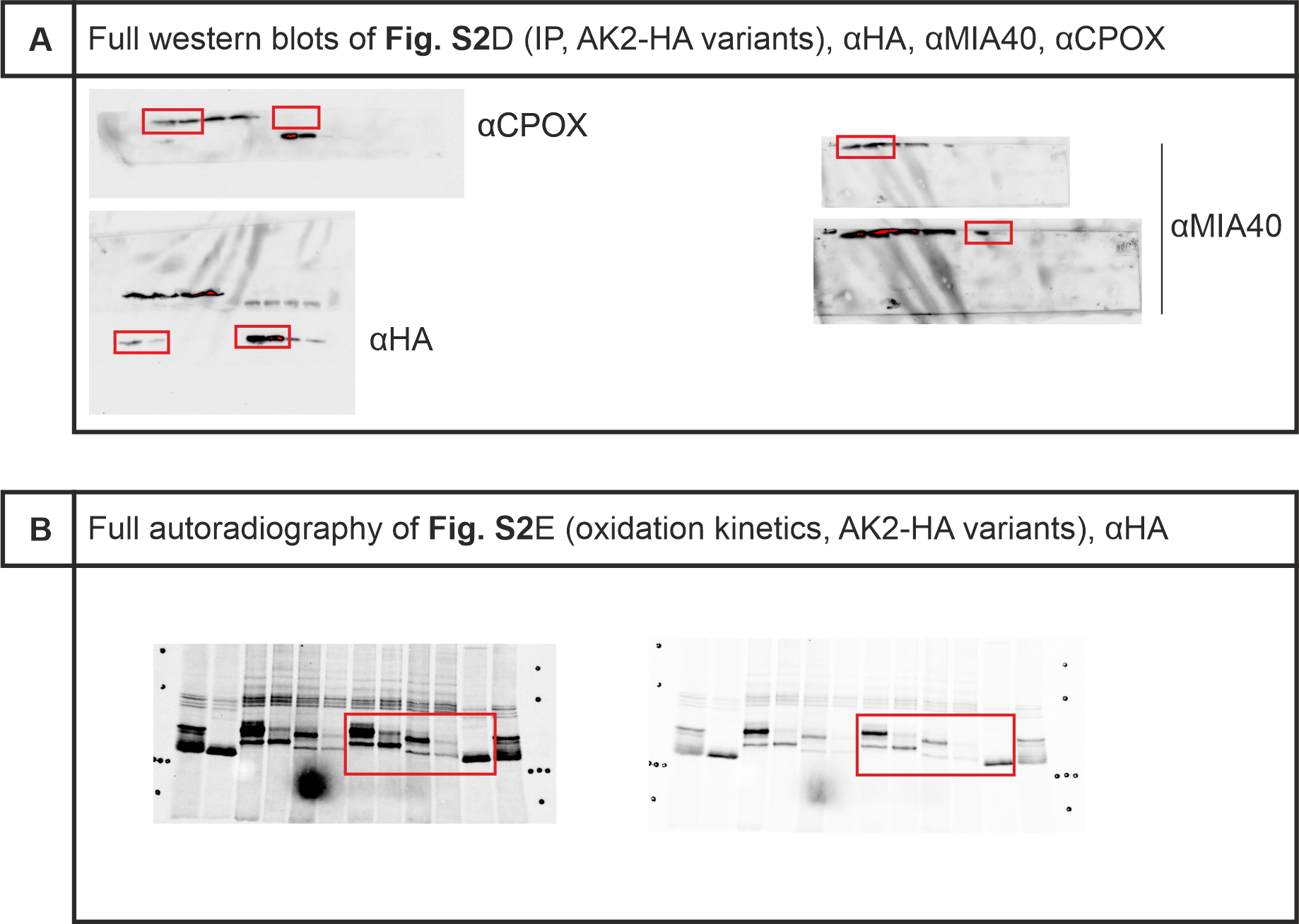

Supplement: Supplementary file 3 — Source Data for Appendix [file EMBJ-39-e103889-s010.zip › Appendix_Figure_Source_Data/Appendix_FigS2_Source_Data/S2-a.png]

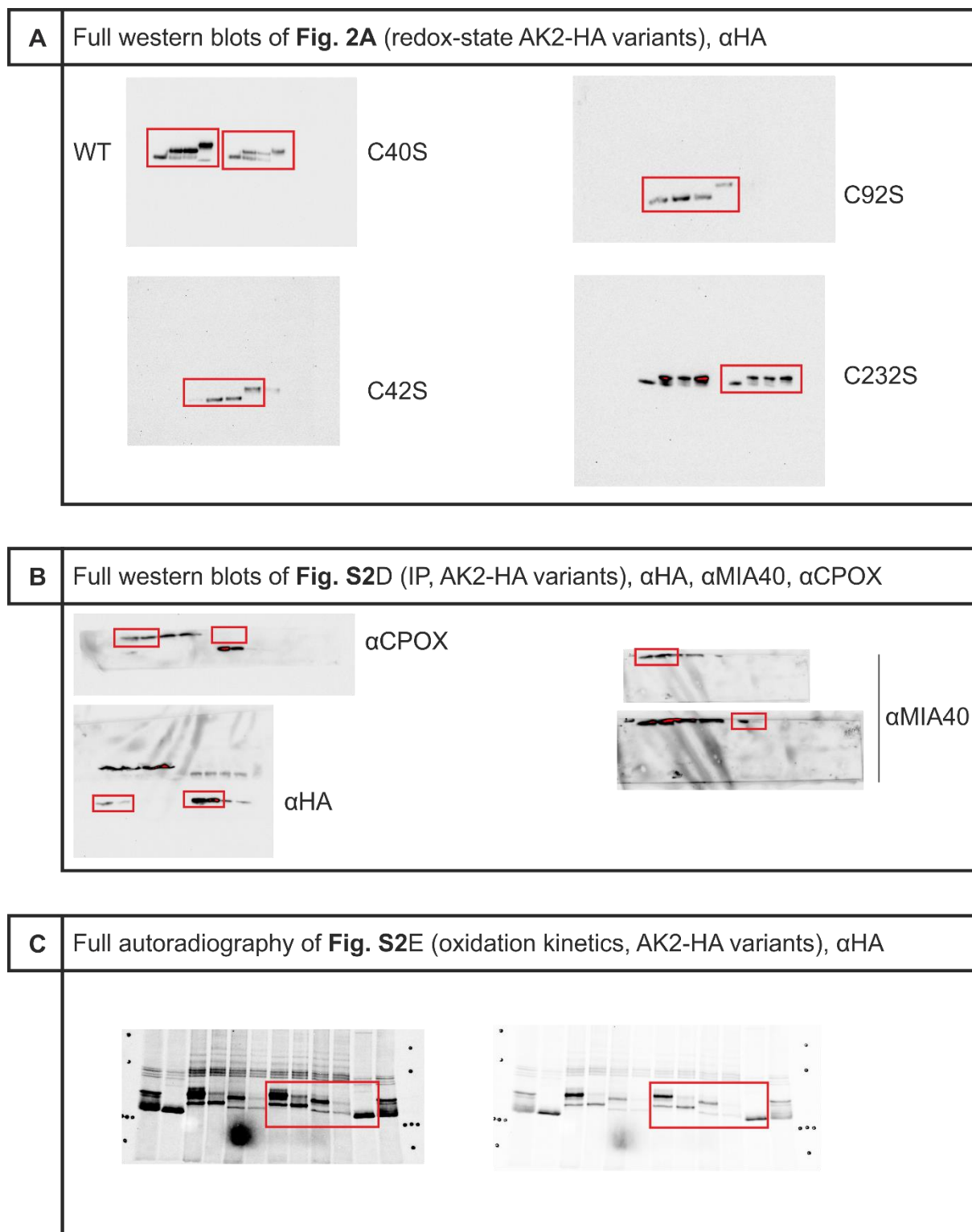

Supplement: Supplementary file 6 — Source Data for Figure 2 [file EMBJ-39-e103889-s004.pdf]

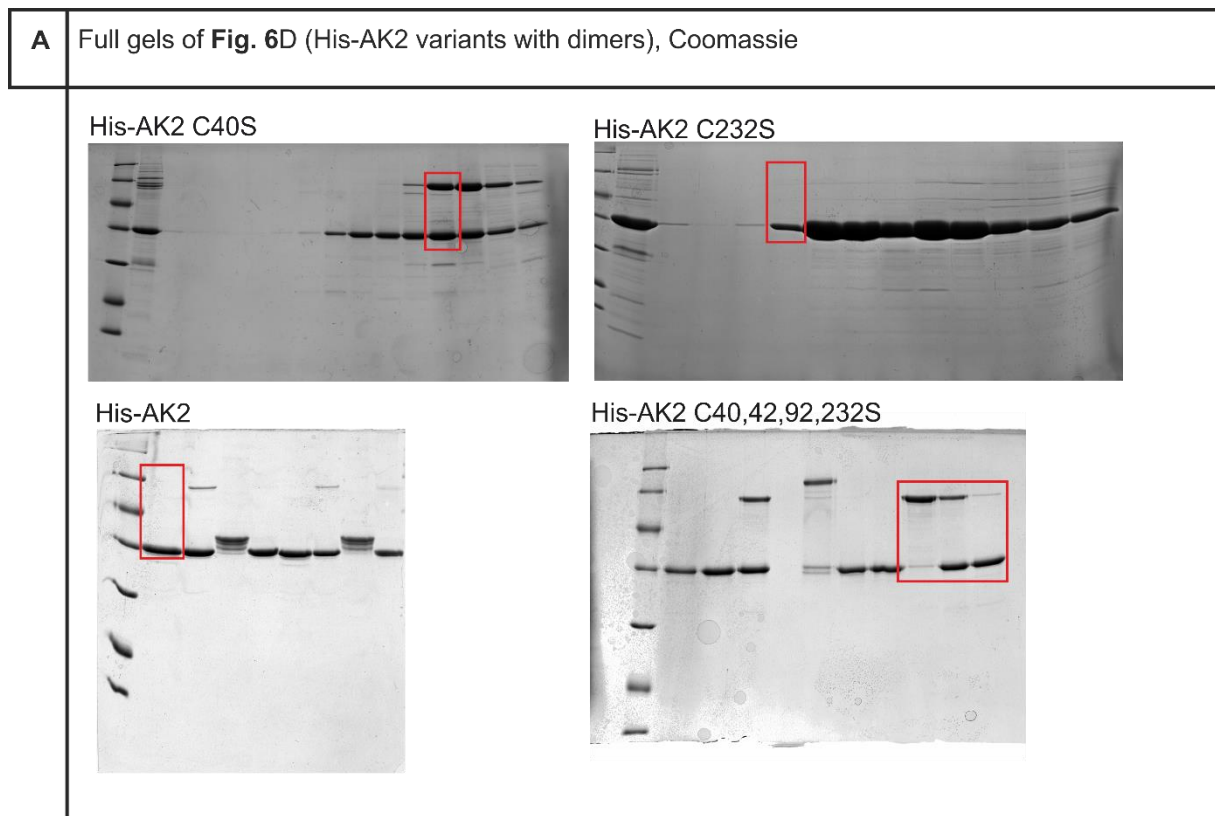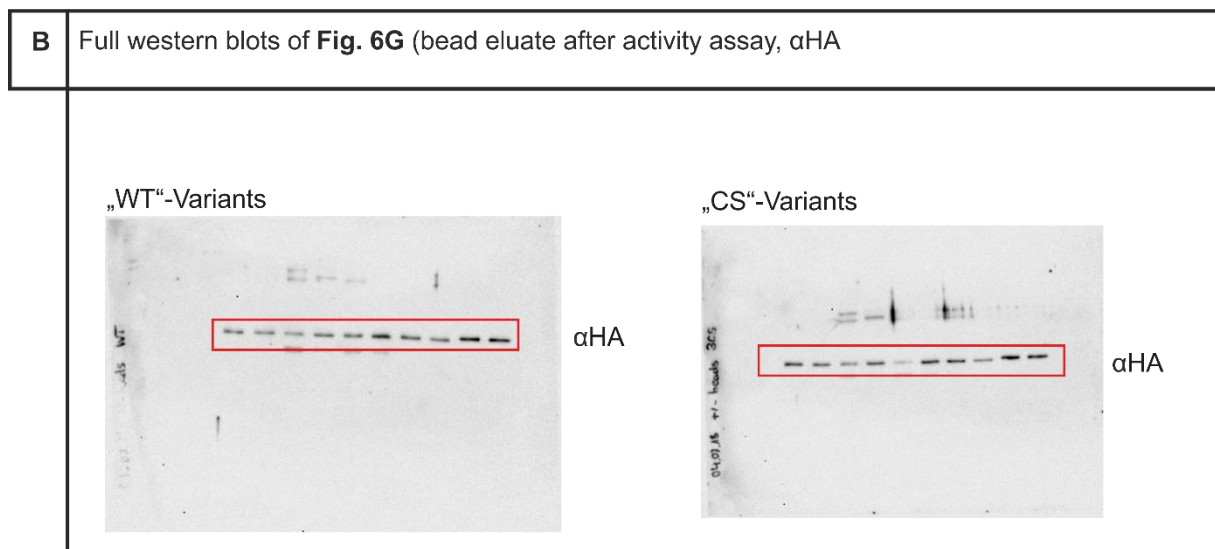

Supplement: Supplementary file 10 — Source Data for Figure 6 [file EMBJ-39-e103889-s008.pdf]
