# Supplementary material for: Proteasomal degradation induced by DPP9‐mediated processing competes with mitochondrial protein import
Source: EMBO J. 2020 Aug 20;39(19):e103889. doi: 10.15252/embj.2019103889 (PMC7527813; doi:10.15252/embj.2019103889)

A Full western blots of **Fig. 1**B (IP MIA40-Strep variants), αAK2, αMIA40, αLDH

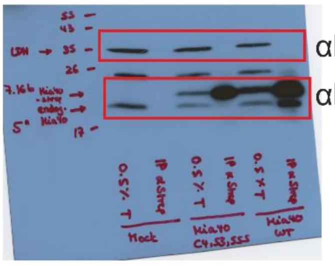

αLDH
αMIA40

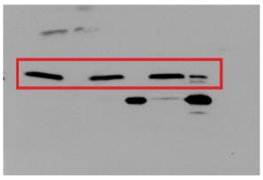

αAK2

B Full western blot of **Fig. 1**C (redox-state, WT), αAK2

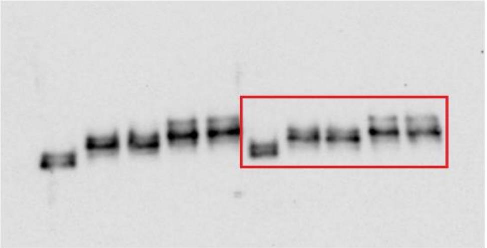

αAK2

C Full autoradiography of **Fig. 1D** (oxidation kinetics), αAK2

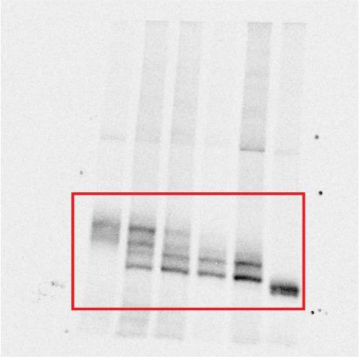

D Full autoradiography of **Fig. 1**E (oxidation kinetics, siRNA MIA40/contr.), αAK2

siRNA MIA40

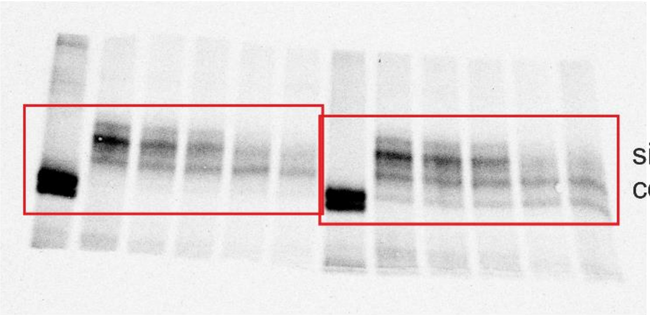

siRNA contr.

E	Full western blot of **Fig. S1**A (redox-state, WT, diff. alkylation variants), αAK2

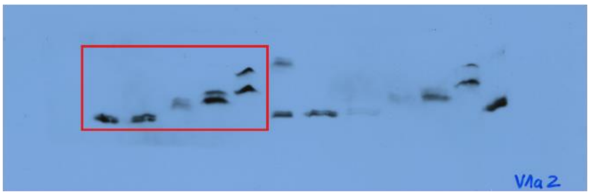

F	Full western blots of **Fig. S1**A (AK2-isoforms, WT,siRNA AK2/contr.), αAK2, αHSP70

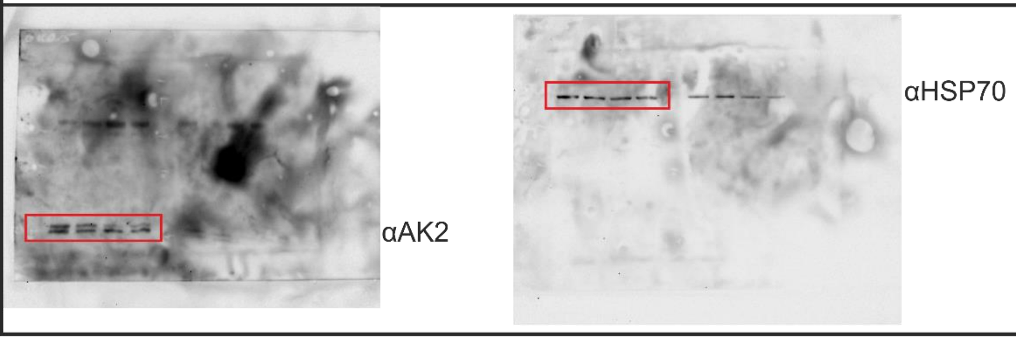

G	Full western blot of **Fig. S1**A (AK2, WT, siRNA AK2/contr.), αAK2

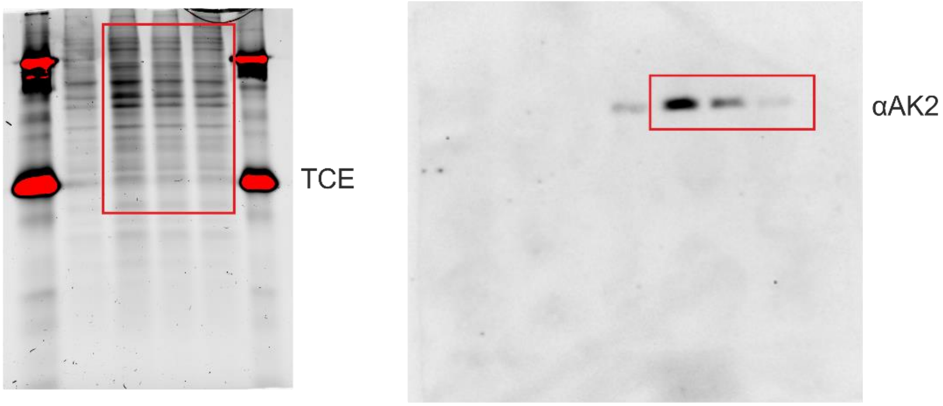

H	Full western blots of **Fig. S1**B (IP AK2-HA),αHA,αMIA40,αHSP70, aCPOX

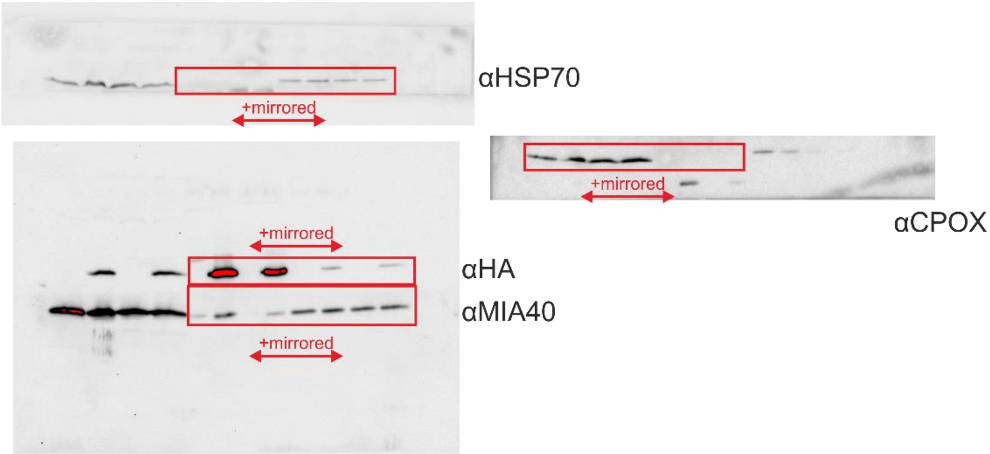

I	Full autoradiography of **Fig. S1**C (IP,re-IP, WT, αAK2, αMIA40)

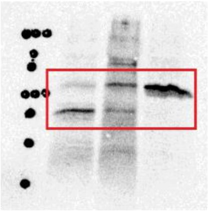

αAK2

J	Full autoradiography of **Fig. S1**D (IP,re-IP, WT, αHA, αMIA40)

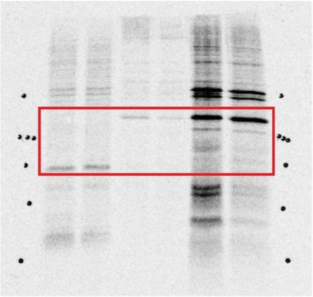

K	Full western blot of **Fig. S1**E (IP aStrep, aAK2, aStrep, aSMAC)

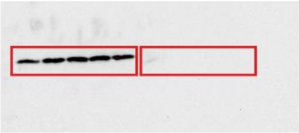

αSMAC

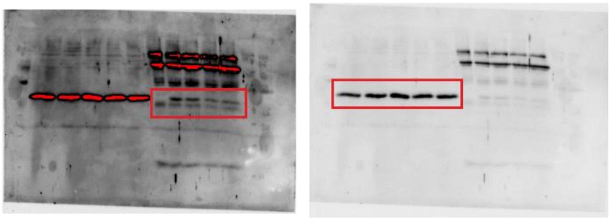

αAK2

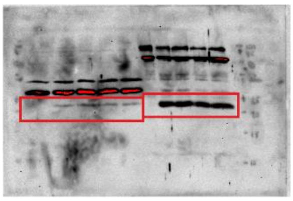

αStrep

L	Full western blot of **Fig. S1**F (redox-state, WT, αAK2)

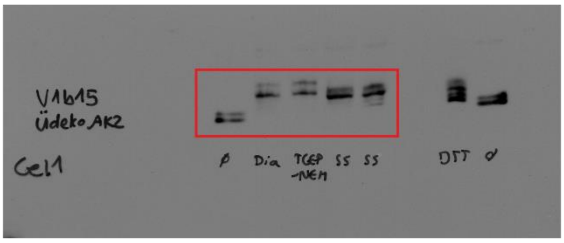

αAK2

**M**	Full western blot of **Fig. S1**G (siMIA, αAK2, αMIA40, αATP5a, αUQCRC2)

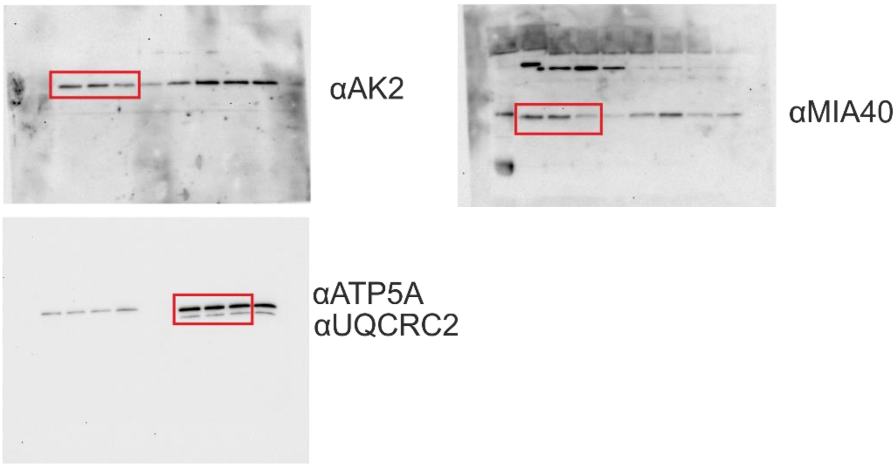

Supplement: Supplementary file 5 — Source Data for Figure 1 [file EMBJ-39-e103889-s003.pdf]
