# Supplementary material for: Proteasomal degradation induced by DPP9‐mediated processing competes with mitochondrial protein import
Source: EMBO J. 2020 Aug 20;39(19):e103889. doi: 10.15252/embj.2019103889 (PMC7527813; doi:10.15252/embj.2019103889)

A | Full western blots of **Fig. 3A** (protein levels, siRNA MIA40, ALR,contr.), αAK2, αMIA40, αALR, αCPOX, αGAPDH

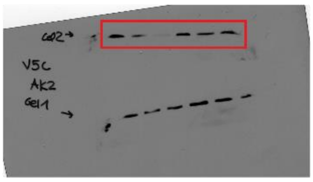

αAK2

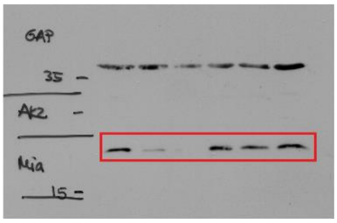

αMIA40

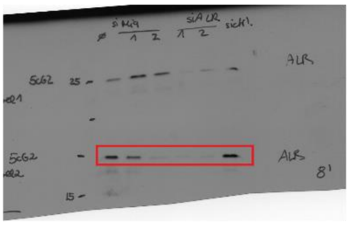

αALR

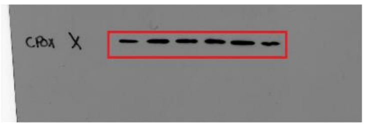

αCPOX

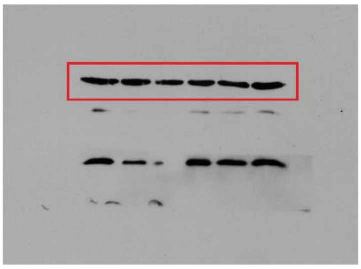

αGAPDH

B | Full autoradiography of **Fig. 3B** (pulse-chase labeling of AK2, siRNA MIA40, contr.), αAK2

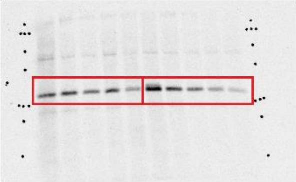

C | Full autoradiography of **Fig. 3C** (proteosomal degradation of AK2 C40,42,92S), αHA

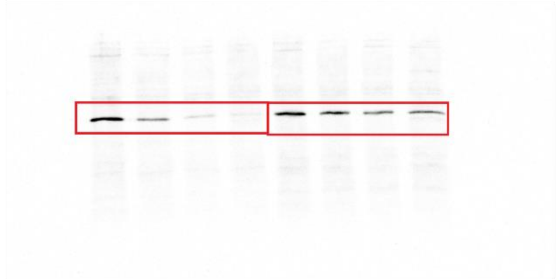

D Full western blots of **Fig. 3D** (proteosomal degradation of AK2 C40,42,92S), αHA, αLDH, αUbiquitin

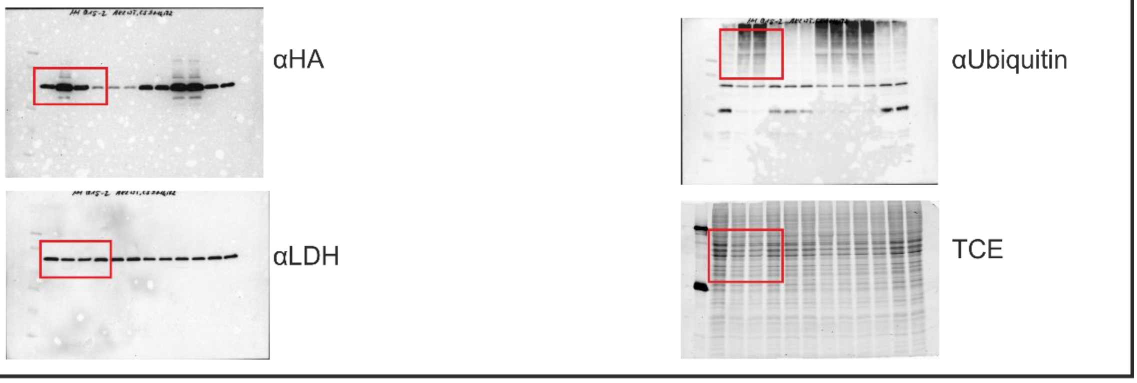

E Full western blots of **Fig. 3E** (proteosomal degradation of AK2 C40,42,92S, siRNA PSMB3/C5, contr.), αHA

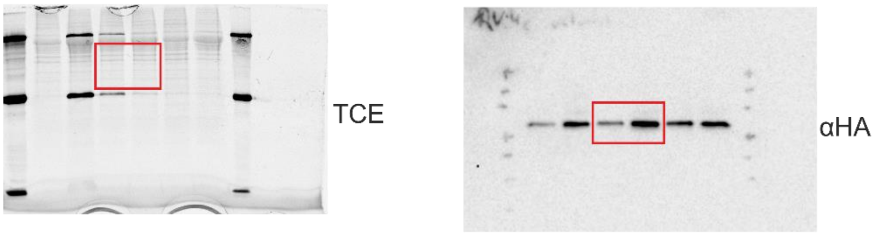

F Full western blots of **Fig. S3**A (protein levels of MIA40-Strep variants), αMIA40, αLDH, αHSP70, αSmac

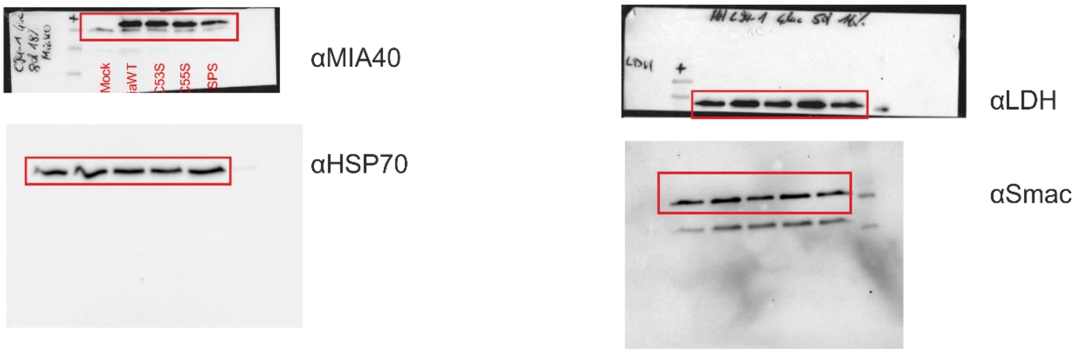

G Full western blots of **Fig. S3**A (protein levels of MIA40-Strep variants), αAK2, αLDHCOX6b1, αCOX17, αNDUFB10, αNDUFS5, αNDUFB7

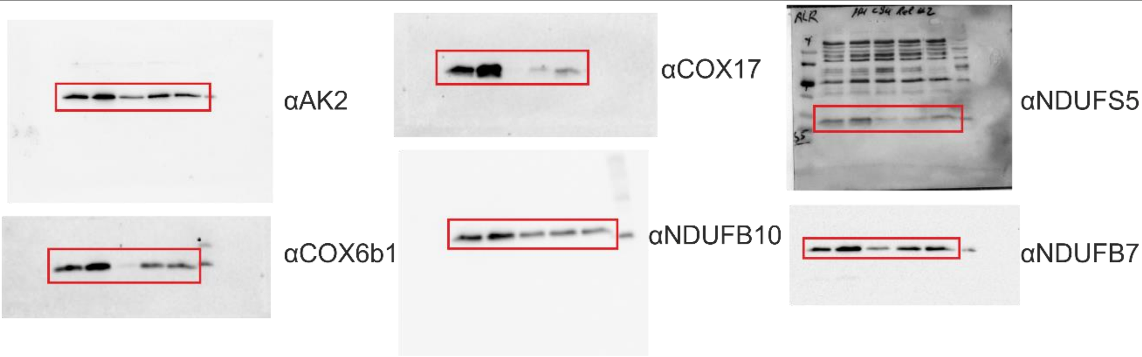

Supplement: Supplementary file 7 — Source Data for Figure 3 [file EMBJ-39-e103889-s005.pdf]
