# Supplementary material for: Proteasomal degradation induced by DPP9‐mediated processing competes with mitochondrial protein import
Source: EMBO J. 2020 Aug 20;39(19):e103889. doi: 10.15252/embj.2019103889 (PMC7527813; doi:10.15252/embj.2019103889)

A Full western blots of **Fig. 4B** (protein levels of AK2 C40, 42, 92S,siRNA DPP8/DPP9/ DPP8+DPP9/contr., proteasomal inhibition), αHA, αAK2, αDPP8, αDPP9, αLDH

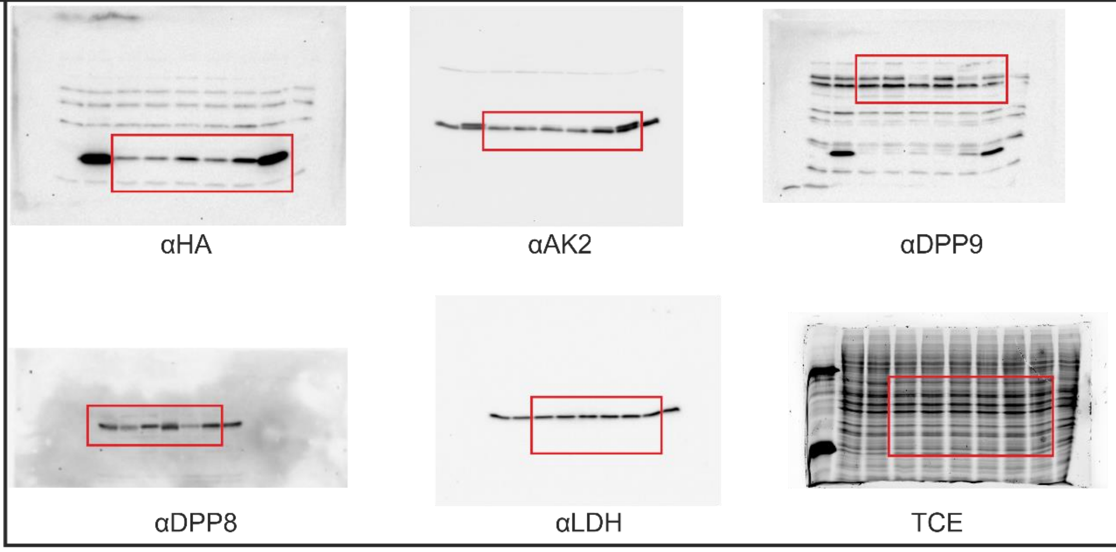

B Full western blots of **Fig. 4C** (protein levels of AK2 C40, 42, 92S, DPP9 inhibition), αHA, αLDH, αDPP9

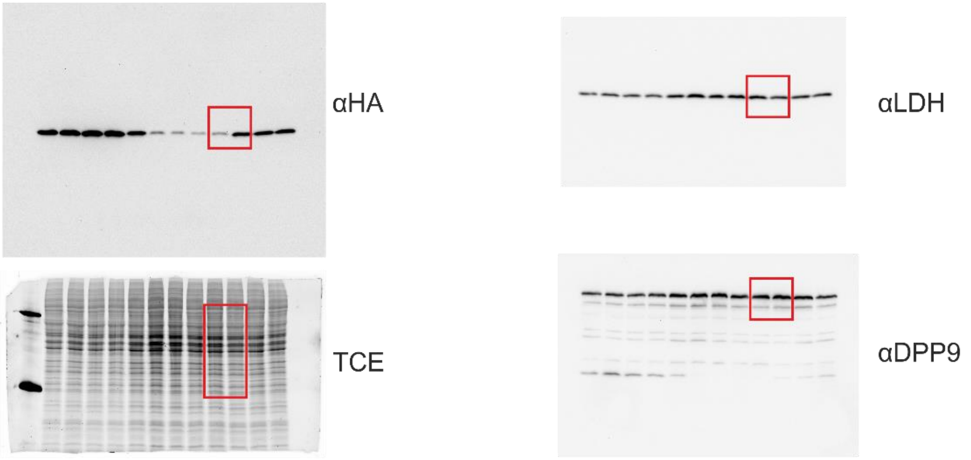

C Full western blots of **Fig. 4D** (protein levels, WT, siRNA DPP9/MIA40/DPP9+MIA40), αAK2, αLDH, αDPP9, αMIA40

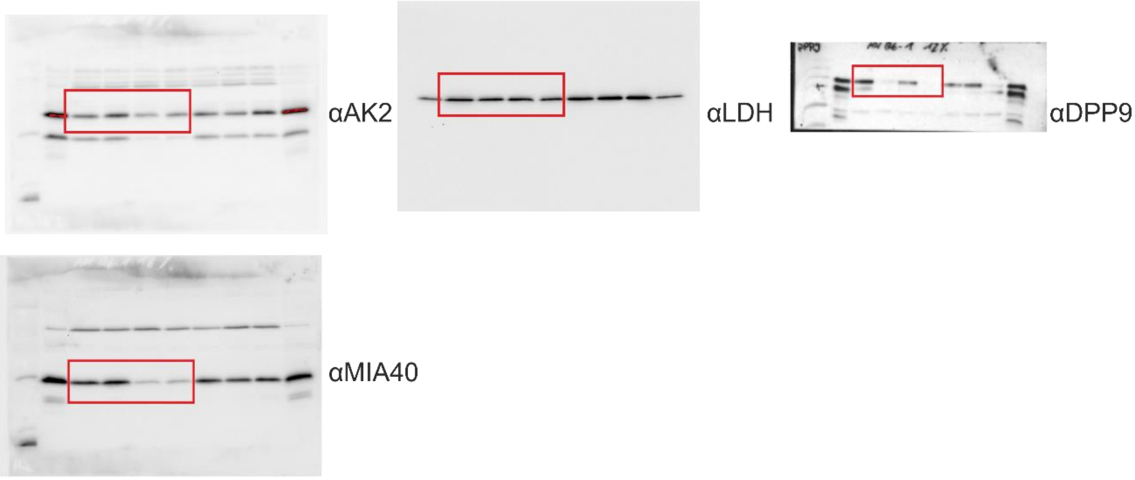

D Full western blots of **Fig. 4E** (protein levels, WT, DPP9 inhibition), αAK2, αDPP9, αLDH

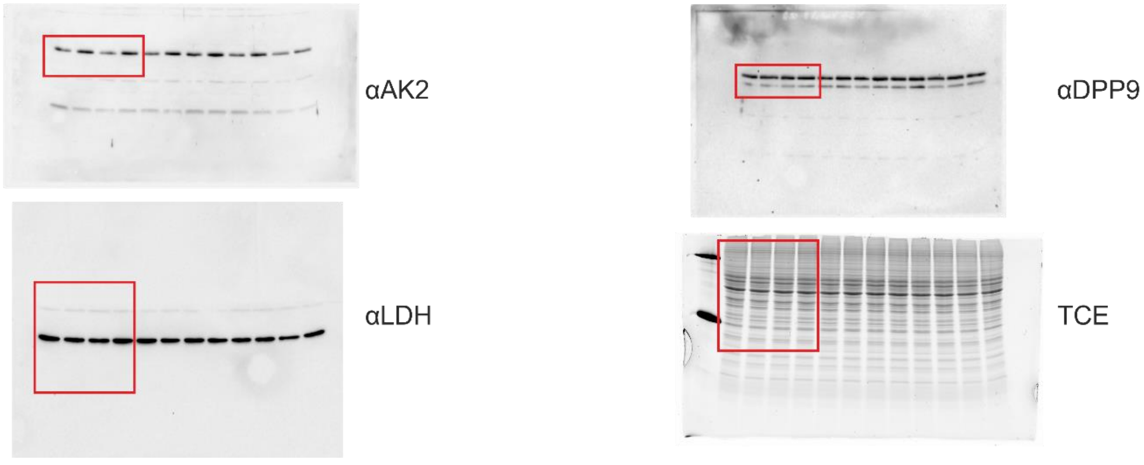

E Full western blots of **Fig. 4F** (protein levels, AK2-HA variants, DPP9 inhibition), αAK2, αDPP9, αLDH

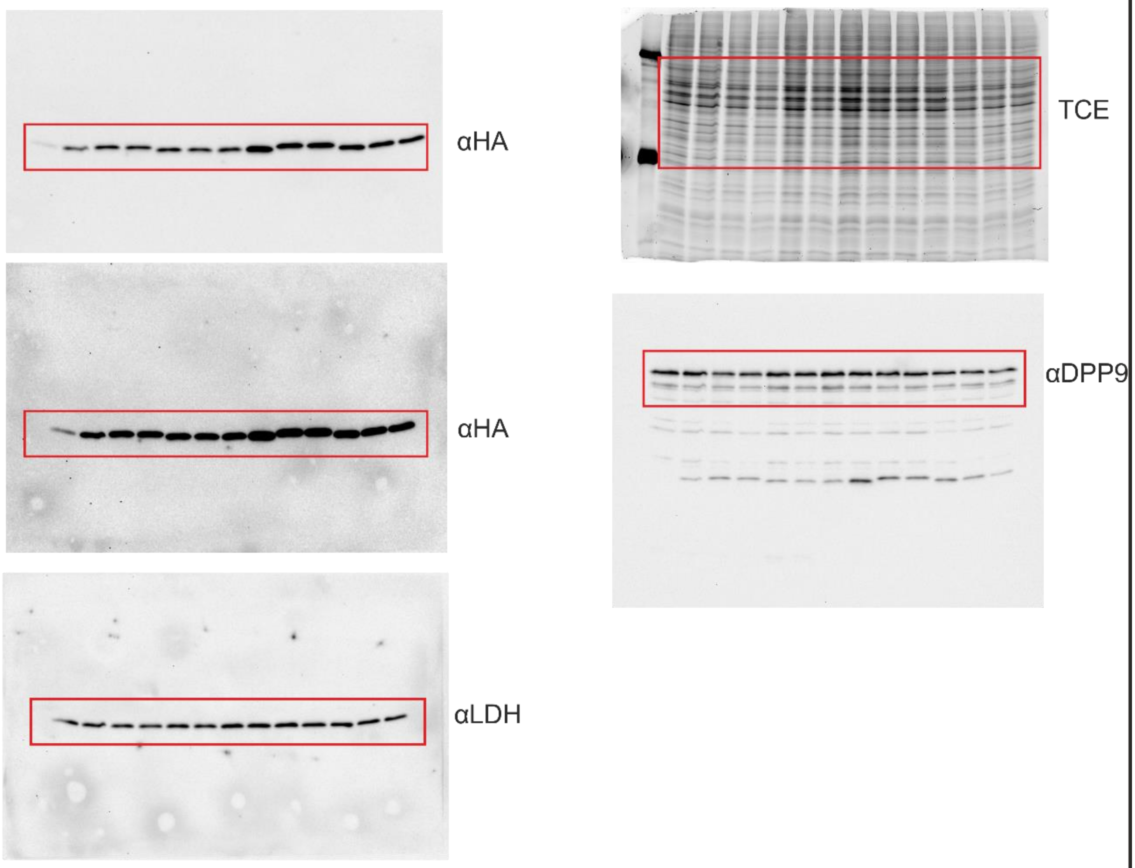

F Full western blots of **Fig. S4B** (protein levels, AK2-HA variants, siRNA DPP9/contr.), αHA, αAK2, αDPP9, αLDH

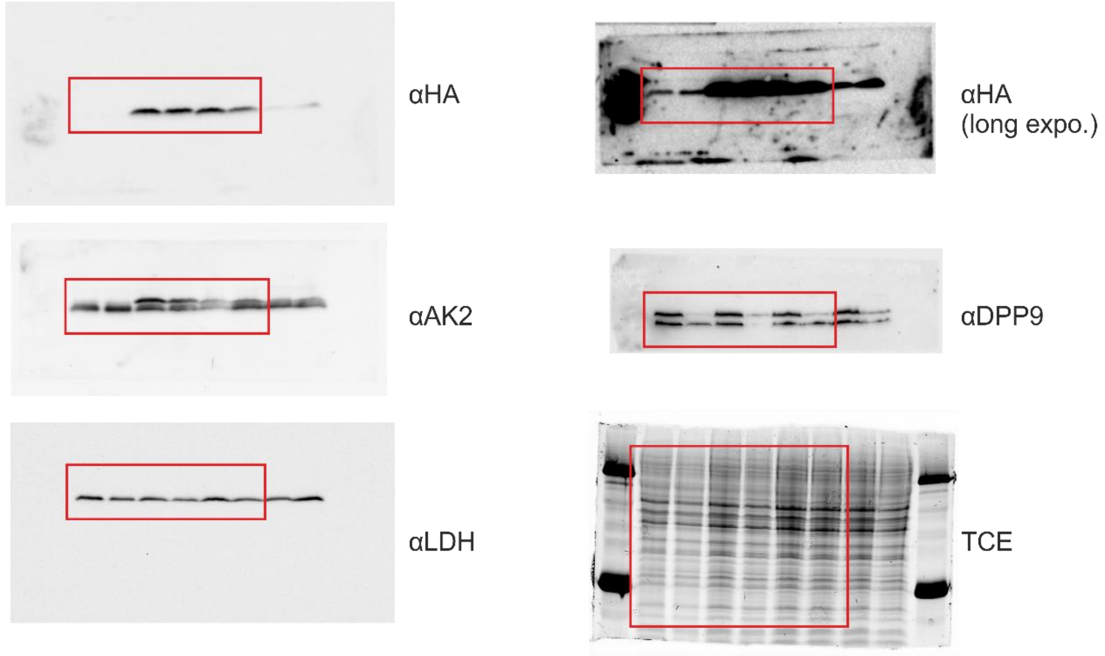

G Full western blots of **Fig. S4C** (protein levels, AK2-HA variants, proteasomal inhibition), αHA, αLDH

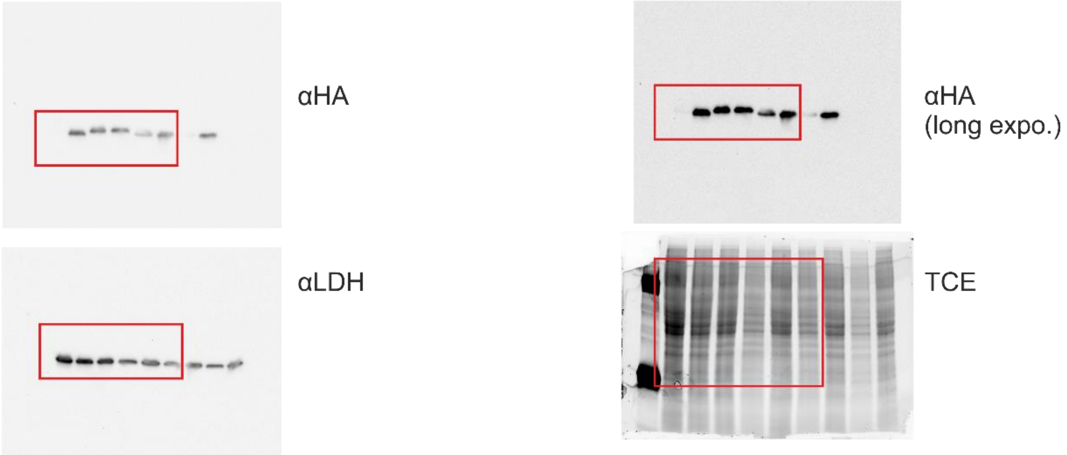

H Full western blots of **Fig. S4D** (protein levels, AK2-HA variants), αHA, αLDH

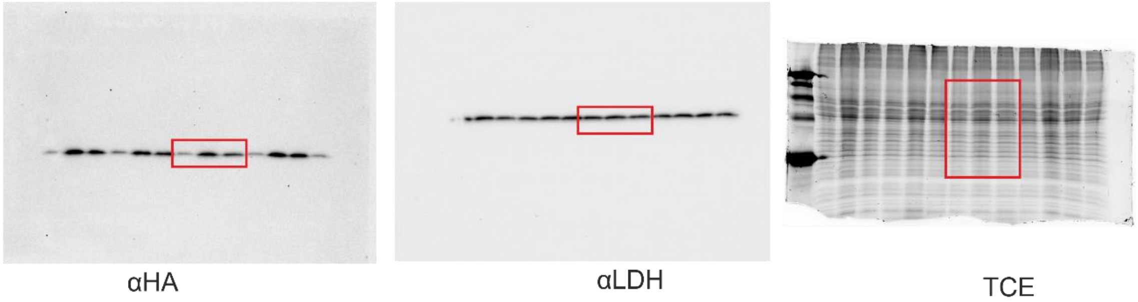

Supplement: Supplementary file 8 — Source Data for Figure 4 [file EMBJ-39-e103889-s006.pdf]
