# Supplementary material for: Proteasomal degradation induced by DPP9‐mediated processing competes with mitochondrial protein import
Source: EMBO J. 2020 Aug 20;39(19):e103889. doi: 10.15252/embj.2019103889 (PMC7527813; doi:10.15252/embj.2019103889)
Supplement: Supplementary file 9 — Source Data for Figure 5 [file EMBJ-39-e103889-s007.pdf]

A Full western blots of **Fig. 5A** (fractionation, WT, DPP9 inhibition), αAK2, αCPOX, αCyt c, αLDH

supernatant

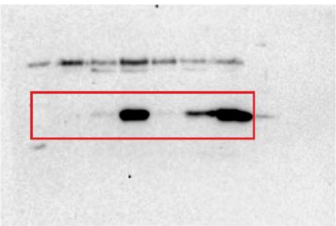

αAK2

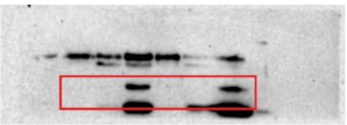

αCPOX

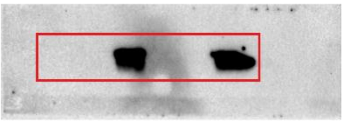

αCyt c

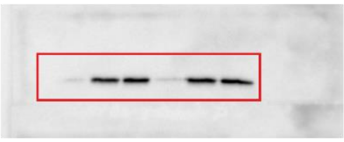

αLDH

pellet

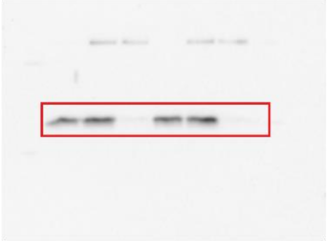

αAK2

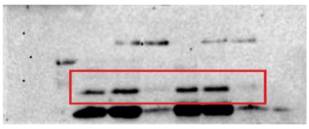

αCPOX

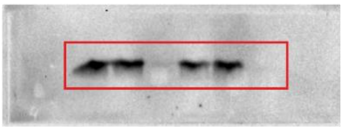

αCyt c

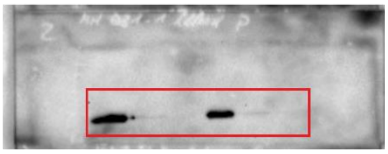

αLDH

B Full western blots of **Fig. S5B** (proteosomal degradation of AK2 C40S), αHA, αTubulin

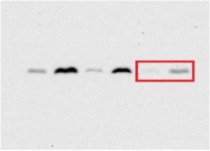

αHA

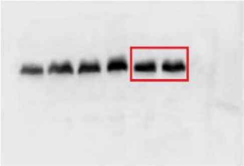

αTubulin

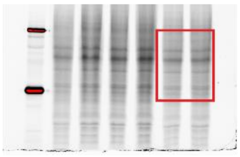

TCE
