# Supplementary material for: Proteasomal degradation induced by DPP9‐mediated processing competes with mitochondrial protein import
Source: EMBO J. 2020 Aug 20;39(19):e103889. doi: 10.15252/embj.2019103889 (PMC7527813; doi:10.15252/embj.2019103889)
Supplement: Supplementary file 11 — Source Data for Figure 7 [file EMBJ-39-e103889-s009.pdf]

A Full western blots of **Fig. 7C** (protein levels of MIA40-substrates, DPP9 inhibition), αHA

COA6-HA

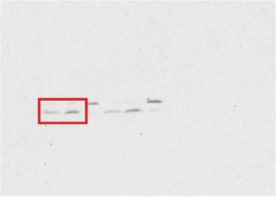

αHA

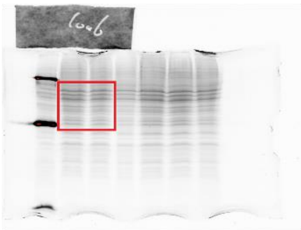

TCE

CHCHD2-HA

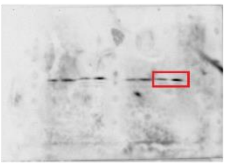

αHA

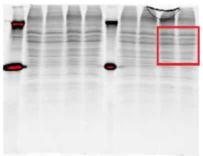

TCE

NDUFB10-HA

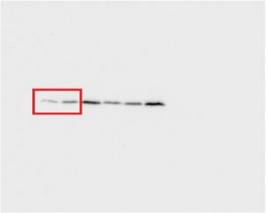

αHA

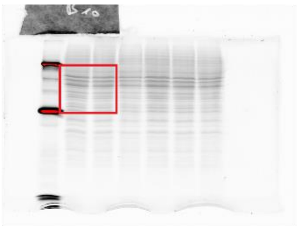

TCE

NDUFS5-HA

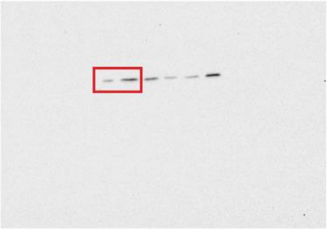

αHA

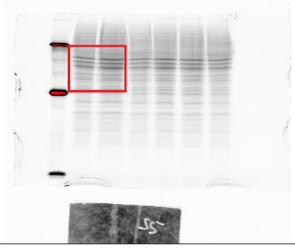

TCE

NDUFA8-HA

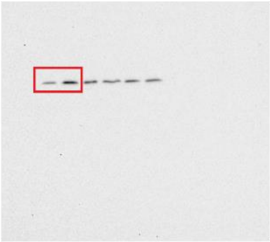

αHA

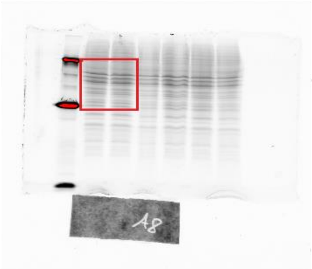

TCE

TIMM9-HA

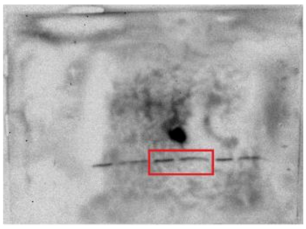

αHA

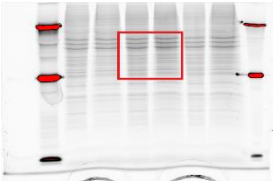

TCE

B Full western blots of **Fig. 7D** (protein levels of NDUFB10 variants, DPP9 inhibition), αHA, αLDH

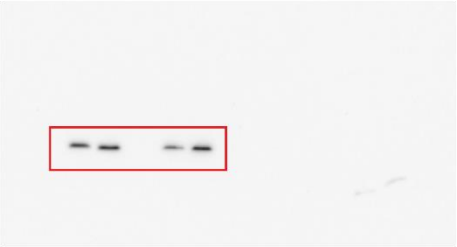

αHA

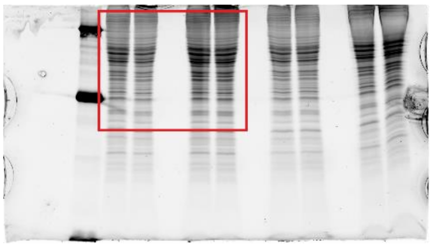

TCE

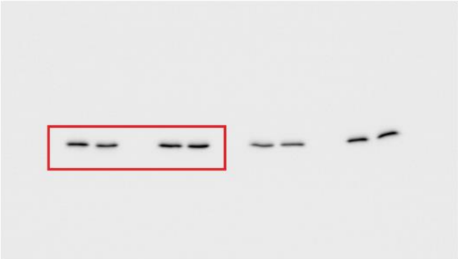

αLDH

C Full western blots of **Fig. 7E** (fractionation of NDUFB10 variants, DPP9 inhibition), αHA, αLDH, αAIFM1

total

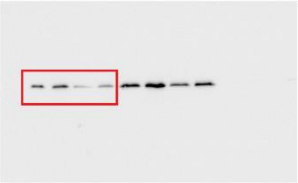

αHA

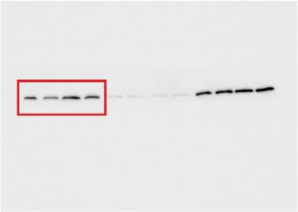

αLDH

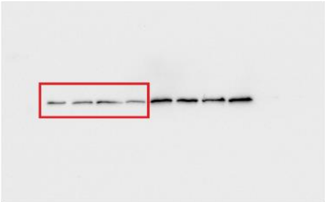

αAIFM1

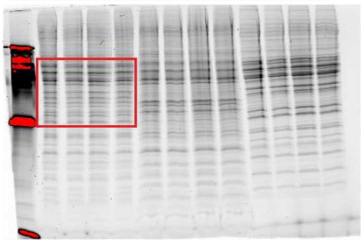

TCE

mitochondria

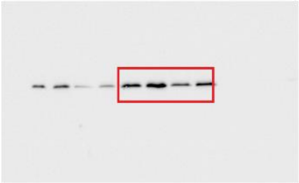

αHA

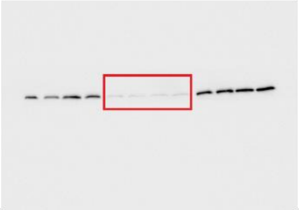

αLDH

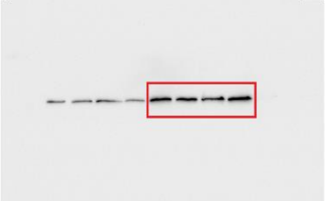

αAIFM1

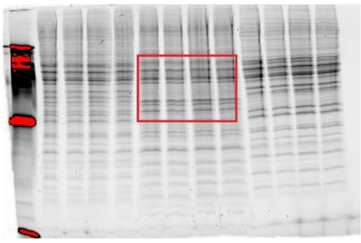

TCE
